# Vitamin D levels do not cause vitamin-drug interactions with dexamethasone or dasatinib in mice

**DOI:** 10.1371/journal.pone.0258579

**Published:** 2021-10-20

**Authors:** Kavya Annu, Kazuto Yasuda, William V. Caufield, Burgess B. Freeman, Erin G. Schuetz

**Affiliations:** 1 Pharmaceutical Sciences, St. Jude Children’s Research Hospital, Memphis, Tennessee, United States of America; 2 Integrated Biomedical Sciences, University of Tennessee Health Science Center, Memphis, Tennessee, United States of America; 3 Preclinical Pharmacokinetic Shared Resource, St. Jude Children’s Research Hospital, Memphis, Tennessee, United States of America; University of Colorado Denver Skaggs School of Pharmacy and Pharmaceutical Sciences, UNITED STATES

## Abstract

Vitamin D_3_ (VD_3_) induces intestinal CYP3A that metabolizes orally administered anti-leukemic chemotherapeutic substrates dexamethasone (DEX) and dasatinib potentially causing a vitamin-drug interaction. To determine the impact of VD_3_ status on systemic exposure and efficacy of these chemotherapeutic agents, we used VD_3_ sufficient and deficient mice and performed pharmacokinetic and anti-leukemic efficacy studies. Female C57BL/6J and hCYP3A4 transgenic VD_3_ deficient mice had significantly lower duodenal (but not hepatic) mouse Cyp3a11 and hCYP3A4 expression compared to VD_3_ sufficient mice, while duodenal expression of Mdr1a, Bcrp and Mrp4 were significantly higher in deficient mice. When the effect of VD_3_ status on DEX systemic exposure was compared following a discontinuous oral DEX regimen, similar to that used to treat pediatric acute lymphoblastic leukemia patients, male VD_3_ deficient mice had significantly higher mean plasma DEX levels (31.7 nM) compared to sufficient mice (12.43 nM) at days 3.5 but not at any later timepoints. Following a single oral gavage of DEX, there was a statistically, but not practically, significant decrease in DEX systemic exposure in VD_3_ deficient vs. sufficient mice. While VD_3_ status had no effect on oral dasatinib’s area under the plasma drug concentration-time curve, VD_3_ deficient male mice had significantly higher dasatinib plasma levels at t = 0.25 hr. Dexamethasone was unable to reverse the poorer survival of VD_3_ sufficient vs. deficient mice to BCR-ABL leukemia. In conclusion, although VD_3_ levels significantly altered intestinal mouse Cyp3a in female mice, DEX plasma exposure was only transiently different for orally administered DEX and dasatinib in male mice. Likewise, the small effect size of VD_3_ deficiency on single oral dose DEX clearance suggests that the clinical significance of VD_3_ levels on DEX systemic exposure are likely to be limited.

## Introduction

The Cyp3a subfamily of enzymes is responsible for the metabolic transformation of many drugs and some endogenous molecules. CYP3A4, an important member of CYP3A family in humans, can metabolize at least 50% of prescription drugs, and is found to be highly expressed in drug detoxification organs such as liver and small intestine [1]. Intestinal CYP3A-mediated first-pass metabolism of orally administered substrates such as cyclosporine, midazolam, lovastatin, felodipine, saquinavir, and buspirone is a major basis for their low oral bioavailability [2, 3].

Induction of hepatic and intestinal CYP3A4 is the basis for many drug-drug interactions. CYP3A4 is induced by nuclear hormone receptors in the NR1I family that are each activated by specific ligands: NR1I2/Pregnane X receptor (PXR) (e.g., glucocorticoids, rifampicin) and NR1I3/constitutive activated receptor (CAR) (e.g., phenobarbital-albeit indirectly) [4–6]. We previously determined that CYP3A4 is also induced by NR1I1/Vitamin D receptor (VDR) activated by 1,25 (OH)_2_VD_3_ [7], although this induction occurs primarily in intestinal enterocytes where VDR is highly expressed vs. hepatocytes where it is lowly expressed [8].

Several lines of evidence suggest vitamin D could cause a vitamin-drug interaction (VDI) with CYP3A4. Lindh JD [9] showed in humans that systemic exposure of the immunosuppressant CYP3A4 substrates sirolimus and tacrolimus varied seasonally (lower concentrations in summer), inversely correlating with sunlight exposure and vitamin D levels (high in summer). We showed that human intestinal biopsy CYP3A4 expression varied seasonally with sunlight and vitamin D levels (higher in Spring/Summer and lower in Fall/Winter) and corresponded with a higher systemic midazolam level (indicating lower CYP3A4 activity) in patients dosed in winter vs. summer [10]. Schwartz [11] showed that vitamin D deficient patients given vitamin D supplementation had increased clearance of the CYP3A4 substrate atorvastatin.

Vitamin D deficiency or insufficiency is common in patients with acute leukemias [12]. Approximately 60% of acute lymphoblastic leukemia (ALL) patients at diagnosis are vitamin D insufficient or deficient, with deficiency and insufficiency more common in winter than summer [13]. Vitamin D deficiency in leukemia patients in combination with long term glucocorticoid therapy leads to reduction in bone mineral density (BMD) [14]. Some of these patients are prescribed VD_3_ supplementation to maintain VD_3_ levels and restore BMD [15]. Although intestinal Cyp3a expression can be regulated by VD_3_ and many chemotherapeutic agents are Cyp3a substrates, it has not yet been determined whether vitamin D sufficiency vs. deficiency would affect the systemic concentrations of these chemotherapies. In this study we determined whether vitamin D sufficient vs. deficient mice differ in (a) intestinal expression of VDR target genes, such as Cyp3a, or (b) systemic exposure to the orally administered Cyp3a metabolized leukemia therapies dexamethasone and dasatinib, or (c) dexamethasone’s anti-leukemic efficacy.

## Materials and methods

### Animals

**Animal Welfare**: Association for Assessment and Accreditation for Laboratory Animal Care (AALAC) guidelines were followed for all experimental animal procedures and housing, animal protocol 613-100638-03/20 was approved by the St. Jude Children’s Research Hospital Institutional Animal Care and Use Committee (IACUC). All mice had free access to a continual supply of food and water. All Lab mice are housed in cage systems connected to the automatic watering system receiving fresh, potable, chlorinated, reverse osmosis treated water controlled and monitored by the Watchdog Vivarium Management System that continuously assesses water pressure and flow to make certain water is always available to the animals. Mice are kept under barrier conditions to keep them specific pathogen free. Each animal room and cubicle room in the animal resource center has a separate thermostat and humidistat to control temperature and humidity that is continuously monitored and alarmed. For all non-survival studies mice were observed at least twice-a-week and were euthanized at time points respective to their age to collect blood and harvest tissues (Cyp3a expression) for further analysis or after the completion of the experiment (Pharmacokinetics). **Euthanasia**: Carbon dioxide asphyxiation using a gradual displacement method (fully compliant with the American Veterinary Medical Association Guidelines for the Euthanasia of Animals: Edition 2013) followed by cervical dislocation was used to sacrifice all mice. Assurance of death was assured by pneumothorax or exsanguination. Only trained staff handled the mice, and all animal welfare considerations were made to minimize distress and suffering of the animals.

To generate VD_3_ deficient and sufficient mice, day 4–5 estrus (E5-E6) pregnant C57BL/6J female mice from Jackson Laboratories (Bar Harbor, ME) were kept on the VD_3_ deficient (Harlan catalog no. 5A69, 0 IU of vitamin D) and VD_3_ sufficient diets, (catalog. No. 5BV8, 3.3 IU of vitamin D) to develop vitamin D deficient and vitamin D sufficient mice, respectively. Pregnant dams were maintained on these diets throughout pregnancy, and after parturition, and pups were maintained on the corresponding diets after weaning as described in [16]. The pups were age matched and both male and female, VD_3_ deficient and VD_3_ sufficient mice were used for Cyp3a expression, pharmacokinetic and efficacy studies.

For BCR-ABL survival studies with discontinuous DEX treatment, vitamin D deficiency was generated by placing newly weened pups from C57BL/6J sufficient mice (Jackson Laboratories, Bar Harbor, ME) on VD_3_ deficient and VD_3_ sufficient diets beginning at weaning (a schedule documented to result in vitamin D deficiency within six weeks (16)) in order to eliminate any possibility the vitamin D deficient diet could affect any other systems during fetal development that might impact leukemia development.

To observe the effect of vitamin D status on hCYP3A4 expression, cryopreserved hCYP3A4 transgenic (tg) mouse sperm was used to re-derive the humanized CYP3A4 transgenic mouse model [17]. Human CYP3A4 transgenic mice were obtained from the laboratory of Dr. Frank Gonzalez (Laboratory of Metabolism, Center for Cancer Research, National Cancer Institute, National Institutes of Health, Bethesda). To generate hCYP3A4-Tg VD_3_ deficient and sufficient mice, E5-E6 hCYP3A4-Tg pregnant female mice were kept on VD_3_ deficient and VD_3_ sufficient diets throughout pregnancy and after parturition as described for C57BL/6J mice above [16]. Pups were maintained on the corresponding diets after weaning and were euthanized at 12 weeks of age for tissue collection, blood was collected by cardiac puncture and serum was isolated and stored at -80°C. Tissues such as intestine (duodenum), liver and kidney were harvested and stored in RNA later at -20°C.

Serum samples from all animals in this manuscript were collected but only a few samples were randomly selected across studies and sent to the Veterinary Diagnostic Laboratory at Michigan State University (Lansing, MI) for serum 25-(OH) VD_3_ analysis. Vitamin D deficiency in the mice was confirmed by analyzing serum 25(OH)VD_3_ levels (Table 1) which has a longer half-life (15 days) and is a generally used as a surrogate for serum 1,25 (OH)_2_ VD_3_ which is biologically short lived (~ 15 hours).

**Table 1:** Serum 25(OH)VD3 levels in VD_3_ sufficient and deficient mice.

Strain	VD_3_ group	n	Mean (25-(OH) VD3 nmol/L)	Range or S.D.
C57BL/6	Sufficient	2	324.50	157.00
C57BL/6	Deficient	6	12	5.52
hCYP3A4-tg	Sufficient	4	219	56.02
hCYP3A4-tg	Deficient	4	14.75	2.06

N, number of mice; S.D., standard deviation.

### mRNA quantification by real-time PCR

Mouse intestine (duodenum), liver and kidney tissues stored in RNA later at -20°C were used for RNA isolation. Trizol (Ambion Life technologies, CA, USA or Thermo Fisher scientific, CA, USA) was used to extract RNA according to the manufacturers protocol. Sterile Omni tips (OMNI International, GA, USA) were used to homogenize the tissue using Omni tissue homogenizer. 500 ng of RNA was used to generate cDNA using the Thermoscript^™^ RT-PCR system (Invitrogen ThermoFisher, CA, USA). The cDNA was diluted to a total volume of 70 μL. Primers used for the amplification:

mCyp3a11 (F) CTGGGCCCAAACCTCTGCCA; mCyp3a11 (R) TGTGACAGCAAGGAGAGGCGT.
mCyp3a13 (F) TACCCCAGTATTTGATGCAC; mCyp3a13 (R) AGATAACTGACTGAGCCACA.
mCyp3a25 (F) AGAAAGAACGCCTTGCTTCA; mCyp3a25 (R) TTGGGCAGAGTTCTGTCA.
mCyp3a44 (F) TTGTGGAGGAAGCCAAAAAGTTT; mCyp3a44 (R) TGAGAAGAGCAAAGGATCAAAAAAGT
mCyp3a57 (F) AGAAAGATCGCCTTGATTACAACC; mCyp3a57 (R) TCTGTCAATCTCATCCTGAAGTTTC.
mCyp24a1 (F) GTGCGCCAAAAGAGGTGC; mCyp24a1 (R) GTGGTACTCTGCCAGTGTGT.
mMdr1a (F) TGCCACCACGATCGCCGAGA; mMdr1a (R) TCAGCTGCGCCCCTCTCTCA.
mBcrp (F) CCATAGCCACAGGCCAAGT; mBcrp (R) GGGCCACATGATTCTTCCAC.
mMrp4 (F) GGTTGGAATTGTGGGCAGAA; mMrp4 (R) TCGTCGTGTGCTCATTGAA.
hCYP3A4 (F) CACAGATCCCCCTGAAATTAAGCTTA; hCYP3A4 (R) AAAATTCAGGCTCCACTTACGGTG.
mCyp2b10 (F) CTTGCGCCTGCTGGAGCTGT; mCyp2b10 (R) GGTCCAAGGTGGCCCTGTGC.
Gapdh (F) ACCACAGTCCATGCCATCAC; Gapdh (R) TCCACCACCCTGTTGCTGTA.
Villin (F) AGGAAGGAGGAGCACCTGTC; Villin (R) GGACAGCCAGAGAGCTTCAA.

Real-time PCR quantification was performed using SYBR GreenER^™^ qPCR supermix for ABI PRISM Instrument (Life Technologies, Grand Island, NY) according to manufacturer’s instructions. Samples were run at the following conditions: 95°C for 15 min, 40 cycles of 92°C for 30 sec, 60°C for 30 sec, 68°C for 1 min followed by a dissociation step. The relative amounts of mRNAs in each sample were normalized to Villin or Gapdh for intestine samples or Gapdh values for liver and kidney samples to control for quality of the mRNA. Comparative CT (ΔΔCt) method was used to determine quantitative PCR values.

### Dexamethasone pharmacokinetics

#### DEX discontinuous oral administration in drinking water and bioanalysis

8-10-week-old C57BL/6 VD_3_ deficient and VD_3_ sufficient, male (n = 8–13) and female (n = 5) mice /group were treated with the discontinuous DEX regimen as described [18]. 8 mg/L of dexamethasone sodium phosphate (Fresenius kabi LLC, Lake Zurich, IL, USA) was given in drinking water for 3.5 days (ON) followed by removal of dexamethasone (OFF) but administration of 600 mg/L of Sulfamethoxazole (Aurobindo Pharma Inc, Dayton, NJ, USA) and 120 mg/L of trimethoprim (Aurobindo Pharma Inc, Dayton, NJ, USA) for the next 3.5 days. 1 g/L of tetracycline (Sigma-Aldrich Inc, St. Louis, MO, USA) was added during both phases. Assuming that mice drink 5 mL of water per day, the dose they receive would be 1.6–2 mg/kg/day [18]. This discontinuous DEX ON/OFF schedule is then repeated with blood samples collected on day 0 (basal), and after each pulse of DEX i.e., day 3.5, 10.5 and 17.5 during the discontinuous DEX treatment. Blood was collected just before changing the medicated water i.e., evening on days 3.5 and 17.5, and mornings on days 0 and 10.5. After day 17.5, mice were either euthanized or used to repeat the experiment after a 1-month washout period. Blood was centrifuged at 10,000 rpm for 2 minutes and separated plasma was stored on dry ice. At the end of *in vivo* procedures, all plasma samples were transferred from dry ice and stored at –80°C until analysis. DEX plasma concentrations were measured using liquid chromatography with tandem mass spectrometric detection [18].

DEX single oral gavage and bioanalysis. The dosing and sampling scheme to assess DEX PK in mice after a single oral dose was performed as previously described [19]. Briefly, 14-week-old C57BL/6 VD_3_ deficient and VD_3_ sufficient male (n = 5) and female (n = 5) mice/group were given 2 mg/kg dexamethasone sodium phosphate (1.67 mg/kg DEX free acid) dissolved in 0.5% hydroxypropyl-methylcellulose (HPMC) and 1% Tween 80 by oral gavage. Blood was collected via saphenous/retro-orbital vein at time points: 0.25, 0.75, 0.5, 1, 1.5, 2, 4, 8, 16 hours after treatment. Three sets of mice were used for this experiment, where set 1 was sampled at 2,4,8 hr; set 2 at 0.25, 0.75, 1.5 hr; set 3 at 0.5, 1 and 16 hr. Samples were centrifuged at 10,000 rpm for 2 minutes, plasma was removed and stored at -80°C until further analysis. DEX plasma concentrations were measured using liquid chromatography with tandem mass spectrometric detection (LC-MS/MS) [18].

### Dasatinib administration and blood collection

The total plasma pharmacokinetic profile of dasatinib in C57BL/6 VD_3_ deficient and VD_3_ sufficient mice was assessed after single oral gavage of 10 mg/kg of dasatinib. Dasatinib was dissolved in 80 mMol/L sodium citrate buffer pH 3.1 for a total dasatinib concentration of 1 mg/mL and a 10 mL/kg gavage volume. Mice were grouped by VD_3_ status and sex and studied at 8 and 10 weeks of age. Survival saphenous and/or submandibular bleeding of mice was conducted using IACUC-approved methods at 0.125, 0.25, 0.5, 1, 2, 4, 8, 16 and 24 hr. post dose, with 3 mice per time point. Saphenous and submandibular bleeds of mice using a single-use Sarstedt Minivette POCT 50 μL K3 EDTA capillary device are humane methods for obtaining small volumes of blood over multiple time points, preferrable to retroorbital bleeds, and were approved for this study that required blood sampling at multiple time points (three) after dasatinib administration. Each mouse was sampled three times on two separate dasatinib dosing occasions. Blood was collected into the Minivette capillary device, dispensed into a microtube and vortexed to mix the anticoagulant. The tubes were then immediately centrifuged to plasma and stored on dry ice for the remainder of the study. At the end of the *in vivo* procedures, plasma samples were transferred from dry ice and placed at -80°C until analysis.

### Dasatinib bioanalysis

Total plasma dasatinib concentrations were assessed using a sensitive and specific liquid chromatography, tandem mass spectrometry (LC-MS/MS) assay. Dasatinib stock solutions were prepared in acetonitrile and used to spike matrix calibrators and quality controls. Protein precipitation was performed using a 1:4 ratio of plasma to 15 ng/mL erlotinib HCl (St. Jude Compound Management, S00004053, Inventory ID: SJCH0042996, Purity > 95%) in methanol as an internal standard. A 3μL aliquot of the extracted supernatant was injected onto a Shimadzu LC-20ADXR high performance liquid chromatography system via a LEAP CTC PAL autosampler. The LC separation was performed using a Phenomenex Kinetex 2.6 um EVO C18 (100A, 50 x 2.1 mm) maintained at 50°C with gradient elution at a flow rate of 0.5 mL/min. The binary mobile phase gradient began with a linear increase to 100% B in 4.0 minutes. The column was then rinsed for 2.0 minutes at 100% B and then equilibrated at the initial conditions for two minutes for a total run time of eight minutes. Under these conditions, the analyte and IS eluted at 1.81 and 2.21 minutes, respectively.

Analyte and IS were detected with tandem mass spectrometry using a SCIEX API 5500 Q-TRAP in the positive ESI mode with monitoring of the following mass transitions: Dasatinib 488.20 -> 401.00, erlotinib HCl 394.20 -> 250.10.

The experimental bioanalytical runs were all found to be acceptable for the purpose of a singlicate non-GLP, preclinical PK assessment. A linear model (1/X^2^ weighting) fit the calibrators across the 1.00 to 500 ng/mL range, with a correlation coefficient (R) of >0.9058. The lower limit of quantification (LLOQ), defined as a peak area signal-to-noise ratio of 5 or greater verses a matric blank with IS, was 1.00 ng/mL. The intra-run precision and accuracy was < 11.6% CV and 90.6% to 107% respectively.

### Dasatinib and dexamethasone (single gavage) pharmacokinetic analyses

Dasatinib bioanalysis and dasatinib and dexamethasone (single oral dose) pharmacokinetic analysis (S1 Text) were performed by the Preclinical Pharmacokinetic Shared Resource at St. Jude Children’s Hospital using a nonlinear mixed effect modeling approach. Plasma concentration-time (Ct) data in ng/mL for dasatinib or dexamethasone were grouped by individual mouse, Vitamin D status, sex, and/or age, and were analyzed using nonlinear mixed effect (NLME) modeling as implemented in Monolix version 2018R1 (Lixoft SAS, Antony, France). Briefly, parameters and the Fisher Information Matrix (FIM) were estimated using the stochastic approximation expectation maximization (SAEM) algorithm, and the final log-likelihood estimated with importance sampling, all using the default Monolix initial settings, except that 1000 iterations were permitted for estimation of FIM using stochastic approximation. A variety of models were fit to the respective compound’s Ct data, parameterized using apparent clearances, volumes of distribution, and absorption rate constant as needed. These models were assessed for goodness of fit using the -2-log likelihood (-2LL) value, Akaike and Bayesian Information Criterion (AIC, BIC), visual predictive checks, plots of model individual and population predicted vs. observed data, residual plots, and the standard error of parameter estimates. A log-normal inter-individual and inter-occasion parameter distribution was assumed on selected supported parameters, with both on- and off-diagonal elements of parameter covariance matrices tested. Additive and/or proportional error models were tested and implemented as supported. Beal’s M3 method was used to fit any data that were below the LLOQ or above the upper limit of the assay range [20]. The grouping levels were tested as categorical covariates on supported PK parameters, primarily the apparent oral clearance (Cl) for dasatinib, and both Cl and the volume of distribution of the central compartment (V1) for dexamethasone. A covariate effect was considered significant if its addition reduced the -2LL by at least 3.84 units (P < 0.05, based on the χ2 test for the difference in the -2LL between two hierarchical models that differ by 1 degree of freedom). Additionally, Wald test P values were outputted for each covariate effect by the Monolix software.

### Survival study

Murine BCR-ABL Arf^-/-^ Luc^+^ ALL cells were created by Dr. Charles Sherr (St. Jude Children’s Research Hospital) and provided immediately prior to mouse injection by the laboratory of Dr. Mary Relling (St. Jude Children’s Research Hospital) [21]. The cells had been tested for mycoplasma and were authenticated by flow cytometry for cell surface markers, tested for their leukemia-inducing capabilities with serial dilutions of cell numbers (from 2 million down to 2,000 cells), and ensured that the cells with the luciferase constructs produced light when combined with luciferin. Only cells from early passage (P3-P15) were used for these experiments. The St Jude Children’s Research Hospital Institutional Biosafety Committee and IACUC approved the use of these cells for injection into mice. C57BL/6 VD_3_ sufficient (n = 24) and deficient (n = 22) male mice (8–18 wk of age) were anaesthetized using isoflurane and injected iv with 2,000 BCR-ABL Arf^-/-^ Luc^+^ ALL cells on day zero. Body weights were not significantly different at the start of the study (day 0). Starting on day 4, mice were kept ON the discontinuous DEX dosing regimen (described above) where DEX was supplied in drinking water for 3.5 days (ON), and then removed for the next 3.5 days (OFF), and this discontinuous DEX ON/OFF schedule was repeated until the animals had to be euthanized due to leukemia disease burden. Xenogen IVIS-200 (Caliper Life Sciences, Hopkinton, MA) was used to acquire bioluminescent images 5 minutes post-intra peritoneal injection of 200 μL of 100 mg/kg D-Luciferin (Caliper Life Sciences, Hopkinton, MA). Mice were anaesthetized with isoflurane during this procedure. The bioluminescent signal (photons/s) from a fixed region of interest (ROI) is used to monitor the disease progression twice every week. Images acquired here were analyzed using Living Image 3.1 software (Caliper Life Sciences), where whole body luciferase flux measurements (photons/s) were quantified as disease burden. **Animal Welfare:** All efforts were taken to minimize suffering and distress in the survival studies. Mice were observed daily and out of 46 mice used in the survival study, which ended by 61 days, only one mouse was found dead on day 32. All other mice in the survival study were euthanized within hours of reaching the humane end point/end of study = moribund for any reason which was determined based on hind limb paralysis (very typically observed in mice with BCR-ABL ALL), scruffy coat, lethargy or an inability to obtain food or water (SJ IACUC protocol #100468–613). A portion of spleen and hind limb were fixed, paraffin embedded, cut, and slides stained with hematoxylin and eosin and evaluated by a St Jude Pathologist (Dr. Laura Janke) to confirm leukemia was the cause of death [16].

### Data analysis

#### Statistical analysis

GraphPad Prism (version 8) was used for statistical data analysis. Unpaired t-test or Mann-Whitney non-parametric tests were used to determine the significance in gene expression assays. Survival data was analyzed using Kaplan Meier survival analysis and Gehan-Breslow-Wilcoxin test was used to determine significance. Significance was calculated at p<0.05 and the Grubb’s test on GraphPad QuickCals was used to remove any outliers. A 2-way ANOVA was used to test the effect of vitamin D status or time on DEX plasma levels. Further, Sidak’s multiple comparison test was used to analyze the significance of each effect. For the DEX oral gavage PK study, we used a population modeling approach that is more appropriate for individual animals with variable and sparse repeated serial sampling. We tested sex and VD_3_ covariates on the parameter CL/F, that is mathematically proportional to AUC_inf_, as a surrogate for Cmax, these covariates were tested on the volume of distribution parameter V1/F for DEX. Plasma concentration-time (Ct) data in ng/mL for dasatinib were grouped by individual mouse, Vitamin D status, sex, and age, and were analyzed using nonlinear mixed effect (NLME) modeling as implemented in Monolix version 2018R1 (Lixoft SAS, Antony, France). Additionally, Wald test P values were outputted for each covariate effect by the Monolix software.

## Results

### Impact of vitamin D deficiency on murine duodenal Cyp3a and transporter mRNA expression

**Fig 1:**
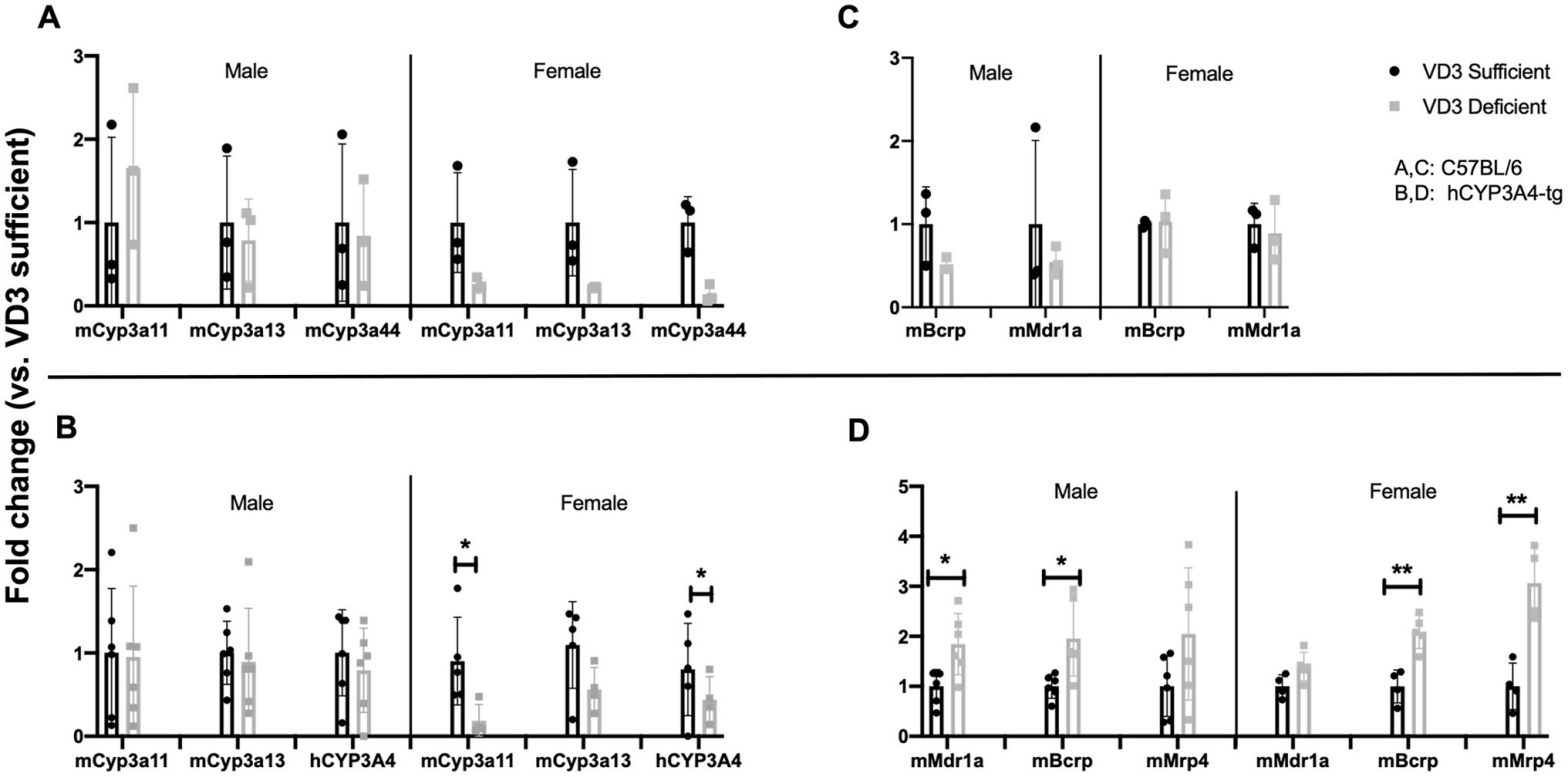
Cyp and transporter gene expression in duodenum of VD_3_ sufficient and deficient mice. Influence of VD_3_ deficiency on duodenal mCyp3a11, mCyp3a13, mCyp3a44 (A,B) and drug efflux transporter mRNA expression (C,D) in 12 wk old C57BL/6 mice (A, C)) and hCYP3A4-tg mice (B,D) (male, n = 3 and female, n = 3/strain). Data are expressed as mean (bars) + SD (error bars) fold change in mRNA expression in deficient mice relative to each mRNAs expression in sufficient mice set as one. Villin was used as an internal control to normalize the target gene expression data. Mann-Whitney nonparametric test on GraphPad was used to determine significance between the groups (**p<0.01, *p<0.05).

Female C57BL/6 VD_3_ deficient mice had lower duodenal mCyp3a11, mCyp3a13 and mCyp3a44 gene expression than VD_3_ sufficient mice, but the difference did not reach statistical significance (Fig 1A), while no differences were observed between male VD_3_ sufficient vs. deficient mice (Fig 1A, S1 Table). To better predict the effect of VD_3_ levels on human intestinal CYP3A4 expression, we used a humanized transgenic mouse containing a bacterial artificial chromosome with the complete human CYP3A4 gene including all of its regulatory sequences [19]. In duodenums of hCYP3A4-tg mice, mCyp3a13 expression trended to be low, both mCyp3a11 and hCYP3A4 mRNA expression was significantly lower in 12-week-old female VD_3_ deficient vs. sufficient mice (Fig 1B, S2 Table). But mCyp3a11, mCyp3a13 and hCYP3A4 expression were not different in duodenums of 12-week-old male VD_3_ deficient hCYP3A4-tg mice (Fig 1B, *p<0.05). Because ABC efflux transporters play a major role in decreasing the oral bioavailability of DEX (Abcb1a/Mdr1a/P-glycoprotein) [22] and dasatinib (Mdr1a, Abcg2/Bcrp) [23] we evaluated their expression. We observed no significant effect of vitamin D status on transporter expression in C57BL/6 mice (Fig 1C), whereas, in hCYP3A4-tg mice, mMdr1a and mBcrp were significantly higher in the duodenums of VD_3_ deficient male mice compared to sufficient mice (Fig 1D, *p<0.05) and mBcrp and mMrp4 were significantly higher in VD_3_ deficient females (Fig 1D, **p<0.01).

### Cyp and transporter expression in liver and kidney of VD_3_ sufficient and deficient hCYP3A4-tg mice

**Fig 2:**
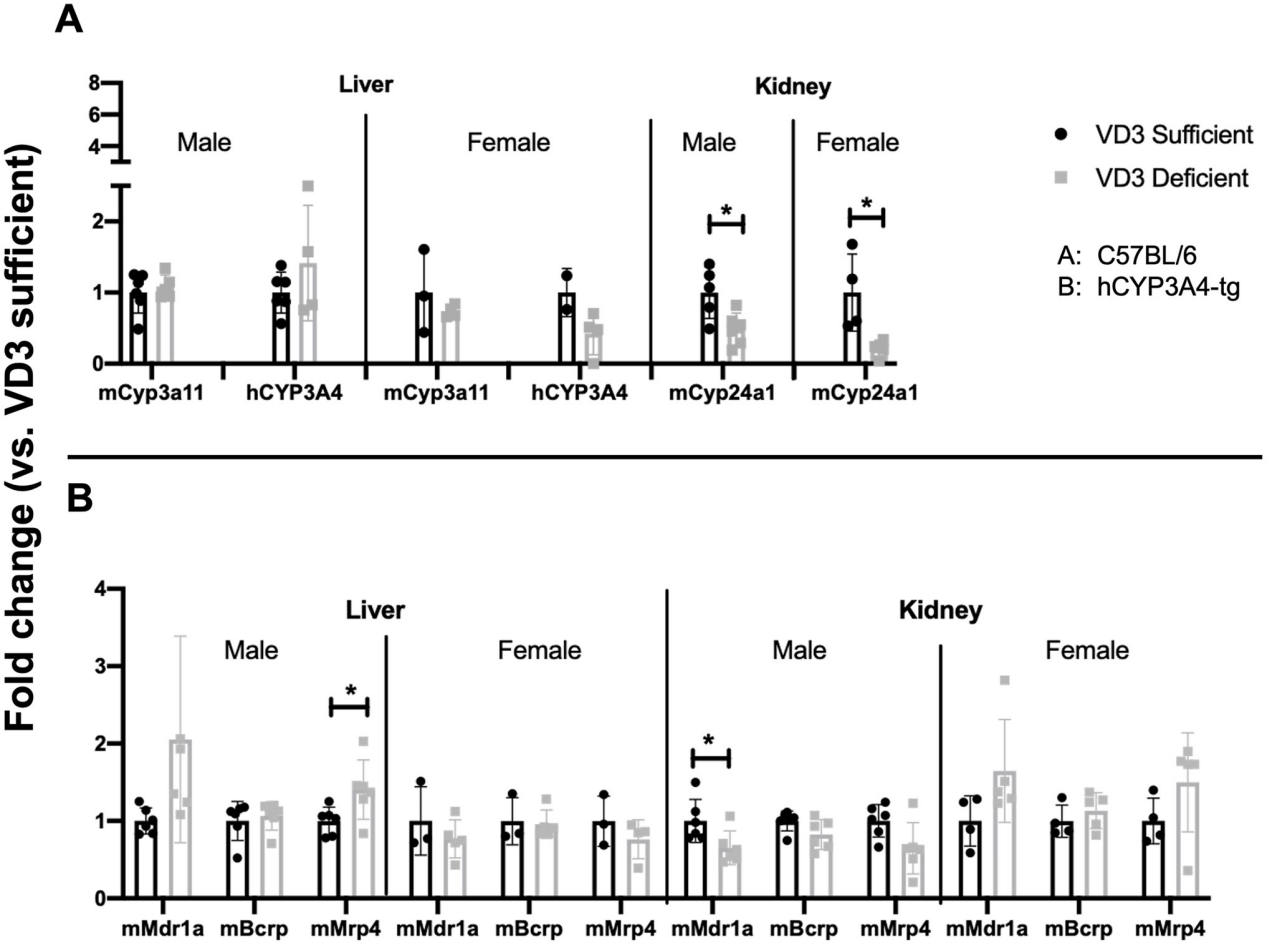
Cyp and transporter gene expression in liver and kidney of 12-week-old hCYP3A4 mice. Comparative expression of hepatic and renal Cyps (A) and efflux transporters (B) in 12-week-old hCYP3A4-tg male and female mice. Data was analyzed and graphed as described in Fig 1 legend.

Because VDR can regulate expression of Cyp and transporters in other tissues important for drug disposition, we examined expression of these detoxification genes in kidneys and livers of mice with different vitamin D levels. VD_3_ status had no significant effect on hepatic Cyp3a11 or hCYP3A4 (Fig 2A, S3 Table). Renal expression of Cyp24a1, a gene highly regulated by VDR [24], was significantly lower in both male and female VD_3_ deficient mouse kidney (Fig 2A *p<0.05). Hepatic Mdr1a trended higher and Mrp4 was significantly higher (Fig 2B, *p<0.05) in male VD_3_ deficient mice (Fig 2B), but no effect of vitamin D status on hepatic transporter expression was seen in female mice. Renal Mdr1a was significantly lower in VD_3_ deficient male mice, while in female mice VD_3_ levels had no significant effect on expression of renal efflux transporters (Fig 2B, S3 Table).

### Plasma dexamethasone concentrations in VD_3_ deficient vs. sufficient C57BL/6 mice

**Fig 3:**
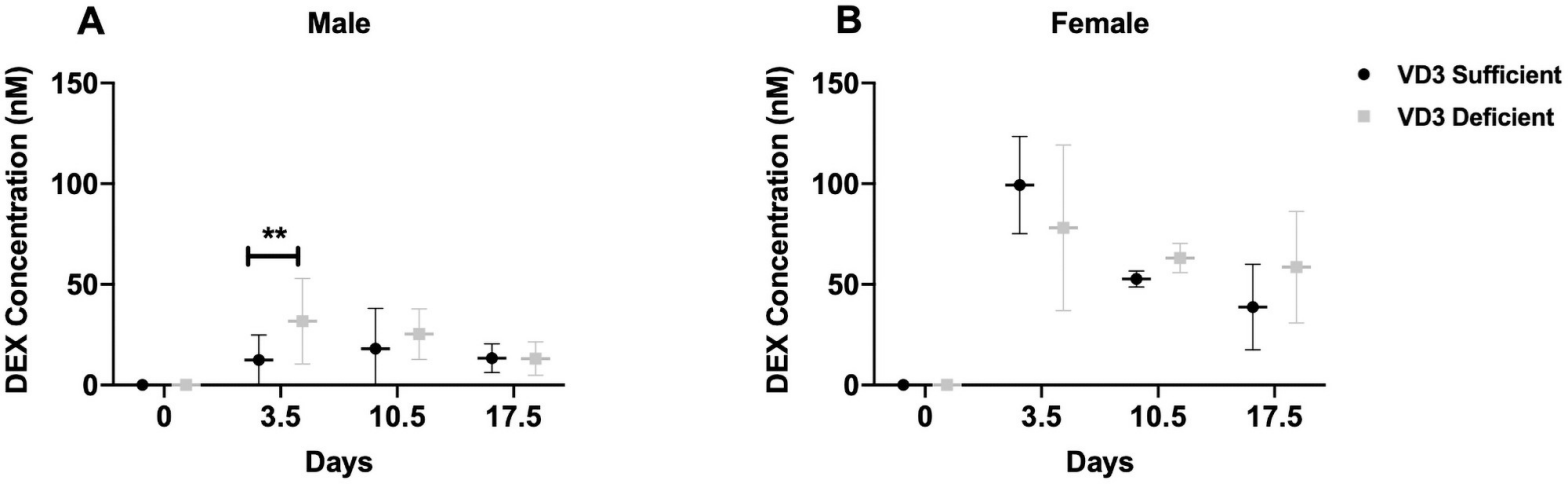
Plasma DEX concentrations in C57BL/6 VD_3_ sufficient and deficient mice on a discontinuous DEX regimen. Plasma DEX concentrations on day 0 (basal), and at the end of each pulse of DEX i.e., days 3.5, 10.5 and 17.5 in (A) male (n = 8-13/group), and (B) female (n = 5) VD_3_ sufficient and deficient mice are plotted as mean + SD. 2-Way ANOVA was used to observe effect of vitamin D status and time (days) on DEX exposure using GraphPad (*p<0.05, ***p<0.001). Sidak’s multiple comparison test (**p<0.01, ****p<0.0001) was used to further analyze the effect of vitamin D status at each time point.

Next, the effect of VD_3_ status on DEX systemic exposure was compared following a discontinuous oral DEX regimen, similar to that used to treat pediatric ALL patients, and that achieves plasma DEX concentrations (2.6–201.5 nM) in mice similar to those achieved in pediatric patients on a discontinuous DEX regimen [18]. Male VD_3_ deficient mice had significantly (Fig 3A, **p<0.01) higher plasma DEX levels compared to sufficient mice with a mean of 31.7 nM (n = 8) and 12.43 nM (n = 11), respectively, on day 3.5, but no difference at later time points. In females (Fig 3B) mean plasma DEX concentrations were not significantly different between VD3 sufficient and deficient mice at any time point.

A 2-way ANOVA was conducted on the effect of vitamin D status or time (days 0, 3.5, 10.5, 17.5) on DEX plasma levels. Overall, effects were significant for both vitamin D status (*p<0.05) and time (***p<0.001) on DEX levels in males. Further, Sidak’s multiple comparison test was used to analyze the significance of each effect, finding at 3.5 days, DEX levels were significantly (**p<0.01) different between VD_3_ deficient versus sufficient mice. In females, time, but not vitamin D status, had a significant (****p<0.0001) effect on DEX levels.

Because there could be some variability in the volume of DEX water consumed, we repeated the oral discontinuous DEX PK study in another 60 mice. The DEX plasma concentrations were below the lower limit of quantification (LLOQ = 5 nM) in a majority of both male (83% Days 3.5+10.5) and female (68% Days 3.5+10.5) VD_3_ *sufficient* mice. In contrast, DEX was measurable in a majority of both female (100%) and male (96.5%) VD_3_ *deficient* mice at the same time points (S4 Table). This suggests, that in VD_3_ *sufficient* mice DEX has been more rapidly cleared.

Because some of the variability in DEX concentrations between mice in all groups could have resulted from the varied consumption of drugged drinking water, the plasma PK profile of DEX was investigated following a single oral gavage (2 mg/kg DEX phosphate, ~ 1.67 mg/kg DEX free acid) in both male and female VD_3_ sufficient and VD_3_ deficient mice. We used a population modeling approach that is more appropriate for individual animals with variable and sparse repeated serial sampling. We tested sex and VD_3_ covariates on the parameter CL/F, that is mathematically proportional to AUC_inf_, and we found that males (regardless of VD_3_ status) had ~25% higher CL/F or lower AUC_inf_. Also, VD_3_ deficiency resulted in a ~25% higher CL/F, or lower AUC_inf_ across both sexes (S1 Fig, S5 Table). This is inverse to what was expected.

As a surrogate for Cmax, these covariates were tested on the volume of distribution parameter V1/F for DEX. Regardless of VD_3_ status, male mice had a 1.43-fold (43%) higher apparent oral volume of distribution (Vl/F) (p = 3.51e-04) than females, and a 1.26-fold higher apparent oral clearance (CL/F) (p = 0.0134), resulting in slightly lower DEX plasma AUC values vs. females.

### Tissue-specific impact of dexamethasone on intestinal and hepatic CYP expression in C57BL/6 sufficient vs. deficient mice

**Fig 4:**
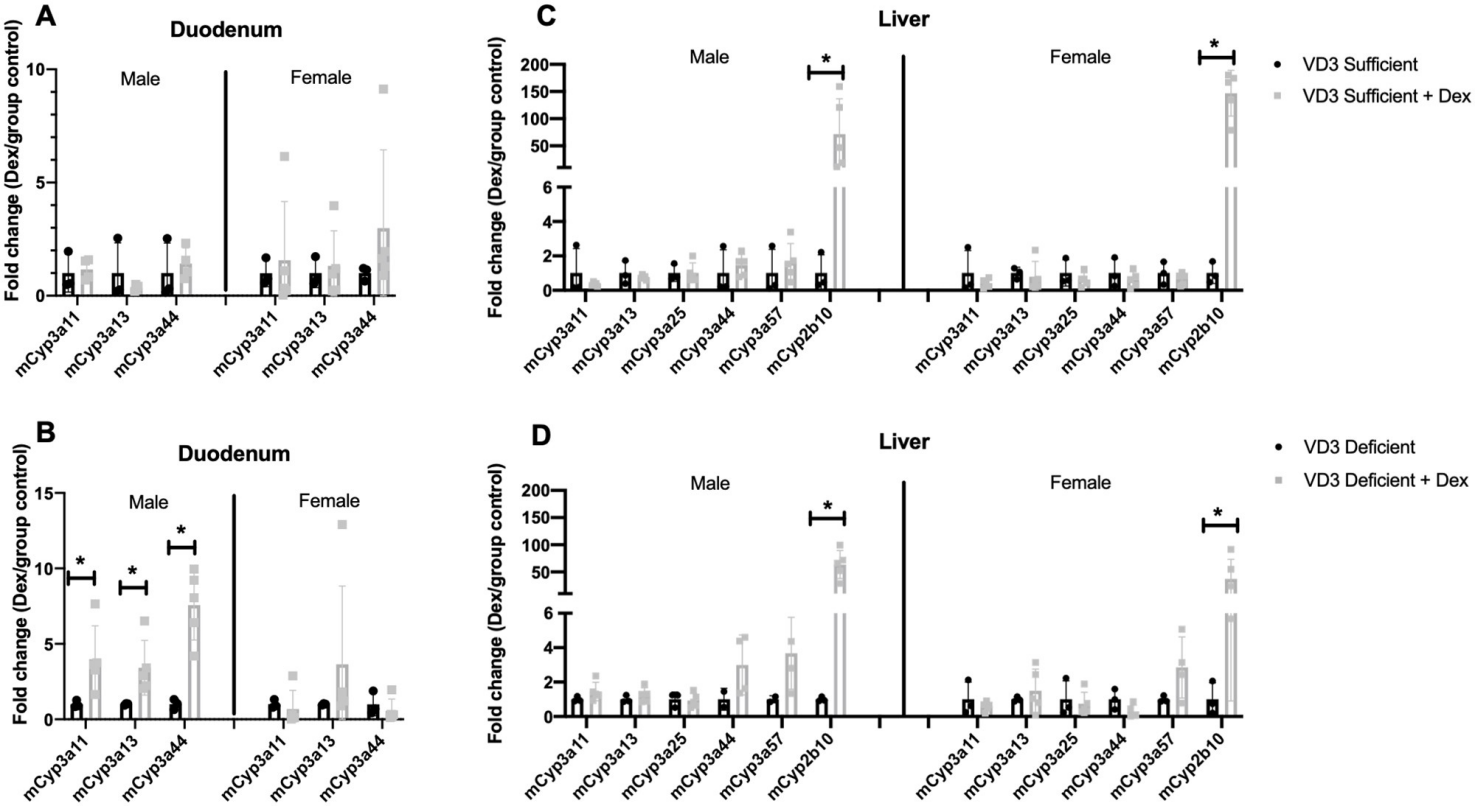
Induction of duodenal and hepatic Cyps in VD_3_ deficient mice after DEX treatment. mCyp3a mRNA expression was analyzed in VD_3_ sufficient and deficient C57BL/6 male mice at time zero and following DEX in drinking water for 3.5 days, n = 3–5 per group: duodenal (A,B) and hepatic (C, D) mCyp expression in VD_3_ sufficient and deficient mice (n = 3–5 per group). Mean fold change + SD are plotted. Gapdh was used as an internal control to normalize the target gene expression data. Mann-Whitney nonparametric test on GraphPad was used to determine significance between the groups (**p<0.01, *p<0.05).

In mice on the discontinuous DEX regimen a significant effect of VD_3_ status on DEX PK was only see at the day 3.5 time-point (Fig 3A). While a number of explanations are possible, we hypothesized that VD_3_ regulated changes in intestinal mCyp3a expression might be affected by prolonged treatment with DEX–another known inducer of mCyp3a. Therefore, we determined whether there was any difference in DEX induction of mCyps in intestine and liver of VD_3_ sufficient vs. deficient mice. mCyp expression was analyzed in duodenum and liver tissue from mice at day zero (no DEX) and on day 3.5 after a pulse of DEX using the oral discontinuous regimen. Duodenal mCyp3a11, mCyp3a13 and mCyp3a44 mRNAs were not induced in male VD_3_ sufficient mice (Fig 4A, S6 Table), but significantly induced (4-6-fold) (Fig 4B, *p<0.05) by DEX in the male VD_3_ deficient mice. In the liver mCyp2b10 was induced at least 50-fold in both VD_3_ sufficient and deficient male and female mice, with no significant induction of hepatic mCyp3as in any mice (Fig 4C and 4D *p<0.05, S7 Table). Thus, there were divergent effects of DEX on induction of mCyp3as and mCyp2b that was both tissue-specific and vitamin D status dependent: DEX induction of mCyp3a was limited to VD_3_ deficient mice in the duodenum, and DEX induction of mCyp2b was observed in liver. A model is proposed in the discussion to account for these differential responses in duodenum vs. liver explaining the approximate tissue specific concentrations achieved and type of receptors/transcription factors that are activated by DEX that could be contributing to the differential effects in gene expression.

### Dasatinib pharmacokinetics (oral gavage) in C57BL/6 mice

**Fig 5:**
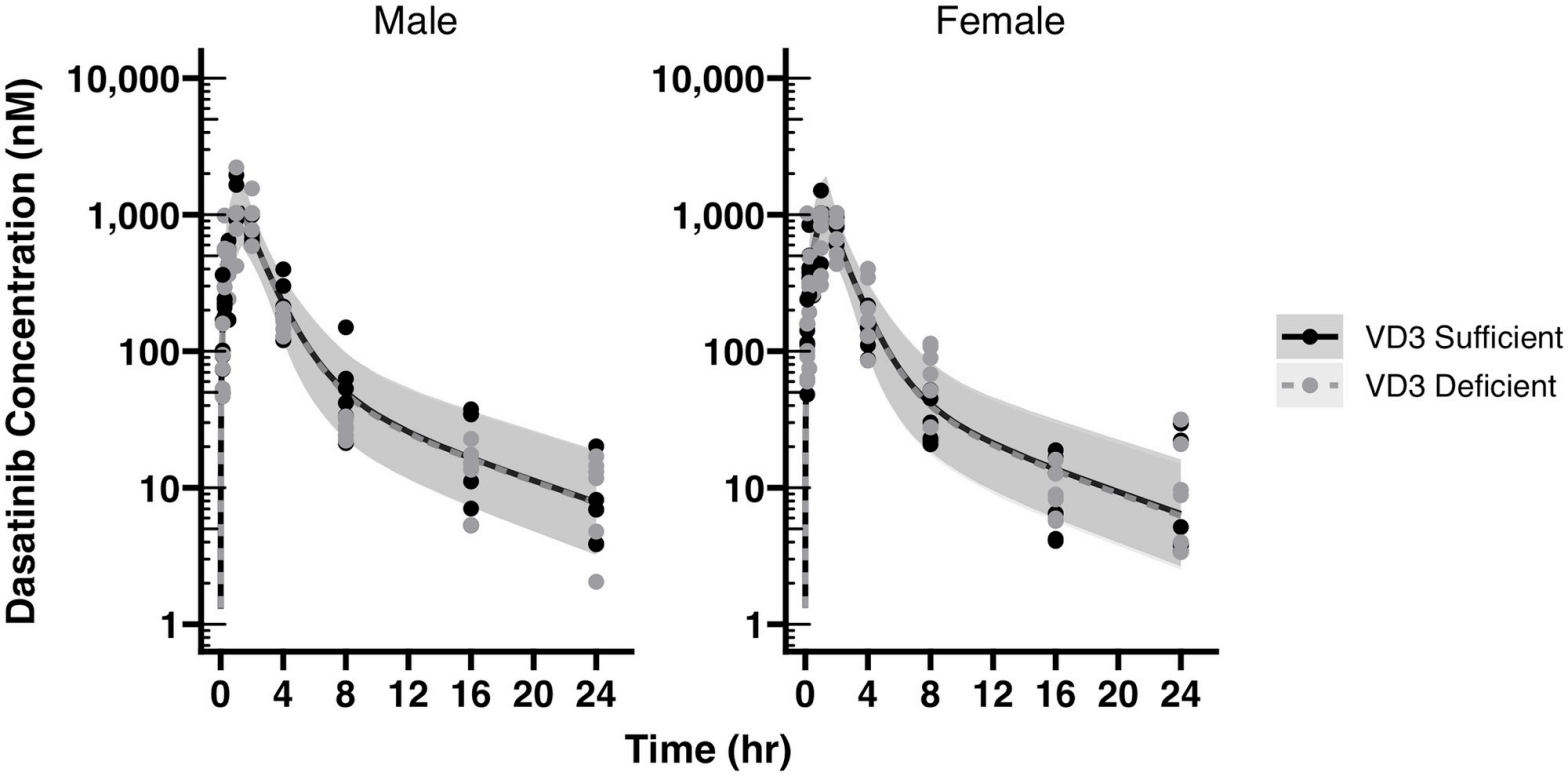
Dasatinib plasma pharmacokinetics in male and female VD_3_ sufficient and deficient mice after a single oral gavage of 10 mg/kg dasatinib. Observed and model predicted dasatinib plasma concentration vs. time (Ct) profiles by sex (panels) and VD_3_ status. The black solid (VD_3_ Sufficient) and gray dashed (VD_3_ Deficient) lines indicate the population model predicted median concentrations, whereas the shaded areas represent the model’s 90% prediction intervals (90% PI) by VD_3_ status. Addition of mouse age did not improve the model fit (p>0.05) and was not incorporated. There were no statistically significant differences in dasatinib apparent oral clearance or AUC by sex (p = 0.905) or VD_3_ status (p = 0.337), as indicated by the overlapping model-fitted profiles.

The plasma PK of dasatinib (a Cyp3a metabolized drug given throughout therapy to treat BCR-ABL+ acute lymphoblastic leukemias) was evaluated after two separate single oral gavage doses of 10 mg/kg to determine the effects of VD_3_ status, sex and age (8 wk vs. 10 wk) on plasma PK using a nonlinear mixed effects PK modeling approach. Blood samples were collected at 0.125, 0.25, 0.5, 1, 2, 4, 8, 16, 24 hr. post-dose with n = 3 mice/group/time point and assayed using LC-MS/MS. There was no influence of VD_3_ status, sex, or age on oral dasatinib PK (Fig 5, *p<0.05, S8 Table).

The oral dasatinib plasma PK in the studied mice was best described using a linear, two-compartment, zero order absorption model, with proportional residual error. Inter-individual variability upon apparent oral clearance (CL/F) and apparent volume of distribution of the central compartment (V1/F) was supported, improved the model fit and performance, as did the off-diagonal correlation between these two parameters. This was defined as the Base model. Inter-occasion variability on either CL/F or V1/F was not supported, resulted in model instability and a poorer model fit. This prevented formal testing of age as a covariate but implies that dasatinib PK is not significantly different between 8 and 10-week-old mice (S1 Text). Vitamin D sufficiency and sex were then tested alone and combined as covariates on CL/F and were also found to be insignificant at the predefined p<0.05 threshold level. Overall, the Base model without any covariates performed the best, and suggested no influence of vitamin D status, sex, or age on oral dasatinib PK in mice.

### Effect of vitamin D status on anti-leukemic efficacy of dexamethasone

**Fig 6:**
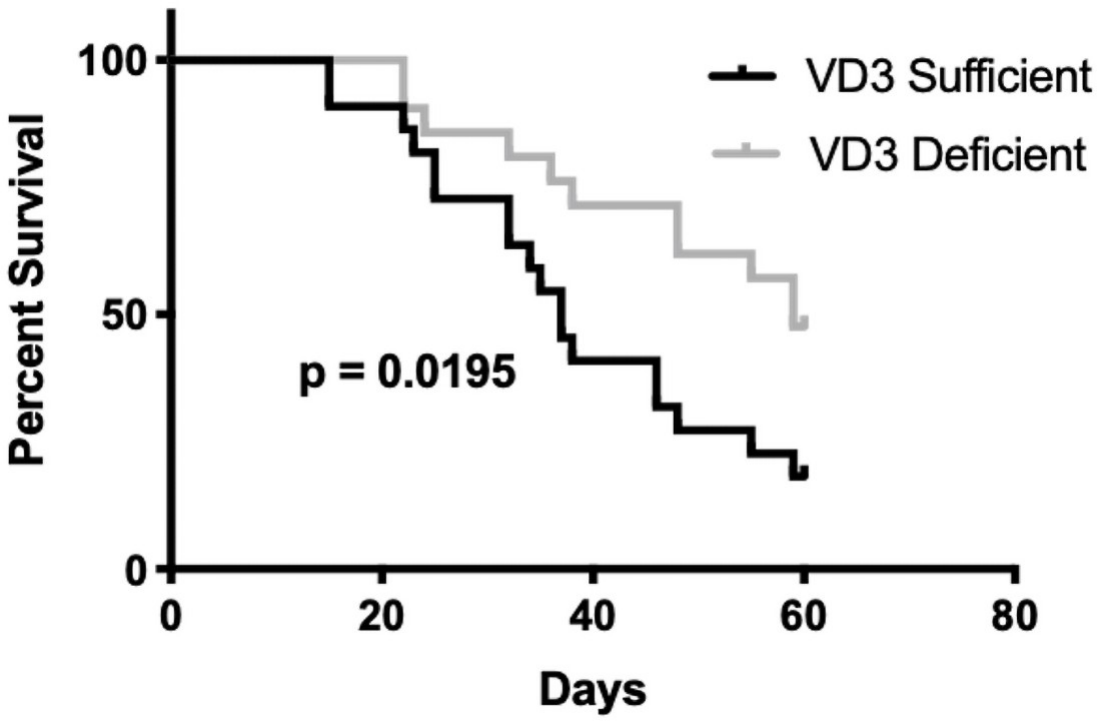
Kaplan-Meier curves for the effect of VD_3_ sufficiency vs. deficiency on survival of mice from BCR-ABL Arf-/- leukemia. Kaplan Meier survival curve plotted for male VD_3_ sufficient and deficient mice with BCR-ABL Arf-/- leukemia and treated with a discontinuous DEX regimen. Prism (version 8) was used to plot and run statistical analysis. The Gehan-Breslow-Wilcoxon test was used to find differences between survival curves of VD_3_ sufficient and deficient groups.

We recently found that VD_3_ deficient mice survived longer with BCR-ABL Arf^-/-^ ALL (acute lymphoblastic leukemia) than sufficient mice in the absence of chemotherapy. We determined that the reason for this paradoxical finding was that vitamin D indirectly affected BCR-ABL ALL progression by remodeling the bone marrow microenvironment, particularly through induction of the chemokine CXCL12 that promoted leukemic blast migration towards the stroma and that directly enhanced blast proliferation [16]. We now compared survival from BCR-ABL Arf^-/-^ ALL between VD_3_ sufficient and deficient mice treated with the oral discontinuous DEX regimen. Leukemia was detected (by imaging the luciferase tagged leukemic blasts by *in vivo* imaging) starting at day 19 in multiple VD_3_ sufficient mice but only in a few VD_3_ deficient mice (S9 Table). VD_3_ deficient mice with DEX treatment survived significantly (Fig 6, S9 Table) longer (median survival time 59 days) compared to the DEX-treated VD_3_ sufficient mice (median survival time 36 days). The proportion of total mice surviving BCR-ABL ALL at various time points (S10 Table) further showed that even with DEX therapy VD_3_ sufficient mice had poorer survival than VD_3_ deficient mice. In sum, even with DEX chemotherapy, VD_3_ sufficient mice have a worse survival outcome than VD_3_ deficient mice from BCR-ABL ALL (Fig 6).

## Discussion

There are at least two clinical trials (Dana Farber and Children’s Hospital of LA: ClinicalTrials.gov Identifier number: NCT01574274 and NCT01317940, respectively) administering vitamin D to insufficient and deficient pediatric acute lymphoblastic leukemia patients to restore VD_3_ sufficiency. Since the biologically active metabolite of VD_3_ (1α,25(OH)_2_D_3_) induces intestinal CYP3A4 in humans and Cyp3as in mice, and since VD_3_ supplementation can alter the pharmacokinetics of orally administered CYP3A4 substrates such as atorvastatin [11], we hypothesized that Cyp3as might be higher in the intestine of VD_3_ sufficient vs. deficient mice and that could lead to increased metabolism, decreased systemic exposure, and decreased anti-leukemic efficacy of the orally administered chemotherapies. DEX is known to be metabolized by CYP3A4 [25, 26] although the exact extent to which CYP3A4 vs, other CYPs metabolize DEX has not been determined. *In vitro* metabolic studies show 98% of dasatinib is metabolized by CYP3A4 in humans [27]. Since both drugs are orally administered to patients during ALL therapy [28, 29], this study sought to determine whether there might be unanticipated VD_3_ interactions with these orally administered CYP3A metabolized ALL therapies.

In the C57BL/6J mouse model, the effect of VD_3_ levels on Cyp3a was intestinal specific—VD_3_ sufficient mice had higher levels of duodenal mCyp3a11 compared to VD_3_ deficient mice while hepatic mCyp3a11 remained unchanged. This result is relevant to human because hCYP3A4 was also significantly lower in the duodenum of the VD_3_ deficient humanized hCYP3A4-transgenic mouse (Fig 1A and 1B) and is what we expect because mCyp3a11 in mouse is considered the mouse orthologue to hCYP3A4 in humans. However, despite the lower intestinal Cyp3a in VD_3_ deficient mice we found serum concentrations of DEX administered by a discontinuous oral regimen were significantly higher only at the earliest (day 3.5), but not later times of DEX therapy in deficient mice. Since we could not be certain that this effect was not due to variation in amount of and time before sampling of drinking DEX water, we administered a single oral gavage of DEX and performed a more sophisticated nonlinear mixed effect modeling analysis finding that VD_3_ *deficiency* resulted in an approximately 25% lower AUC value across both sexes (p<0.05) and that there were statistically significant differences in dexamethasone PK between sexes (S1 Fig, S5 Table). This result is exactly opposite of what would be expected if intestinal Cyp3a was the major driver of oral DEX metabolism and if VD_3_ was causing a Cyp3a-induced vitamin-drug interaction, suggesting other factors such as gut efflux (duodenal Mdr1/Pgp was increased in VD_3_ *deficiency*) (Fig 1) could be reducing DEX bioavailability leading to the apparent DEX clearance being higher in the VD_3_ deficient mice. It is also possible that because the estimated Cgut concentration was so different between discontinuous DEX in drinking water (7–10 μM) vs. single oral gavage (200 μM) that the extent of DEX intestinal metabolism and/or transport differed between the two studies and hence resulted in different results. Nevertheless, despite the statistical significance, the magnitude of the VD_3_ effect was small for both doses of DEX and suggests the clinical significance is likely to be limited given the small effect size. For reference, these differences are within the FDA guidance for therapeutic bioequivalence by plasma AUCs or clearance values, i.e. ± 20%.

**Fig 7:**
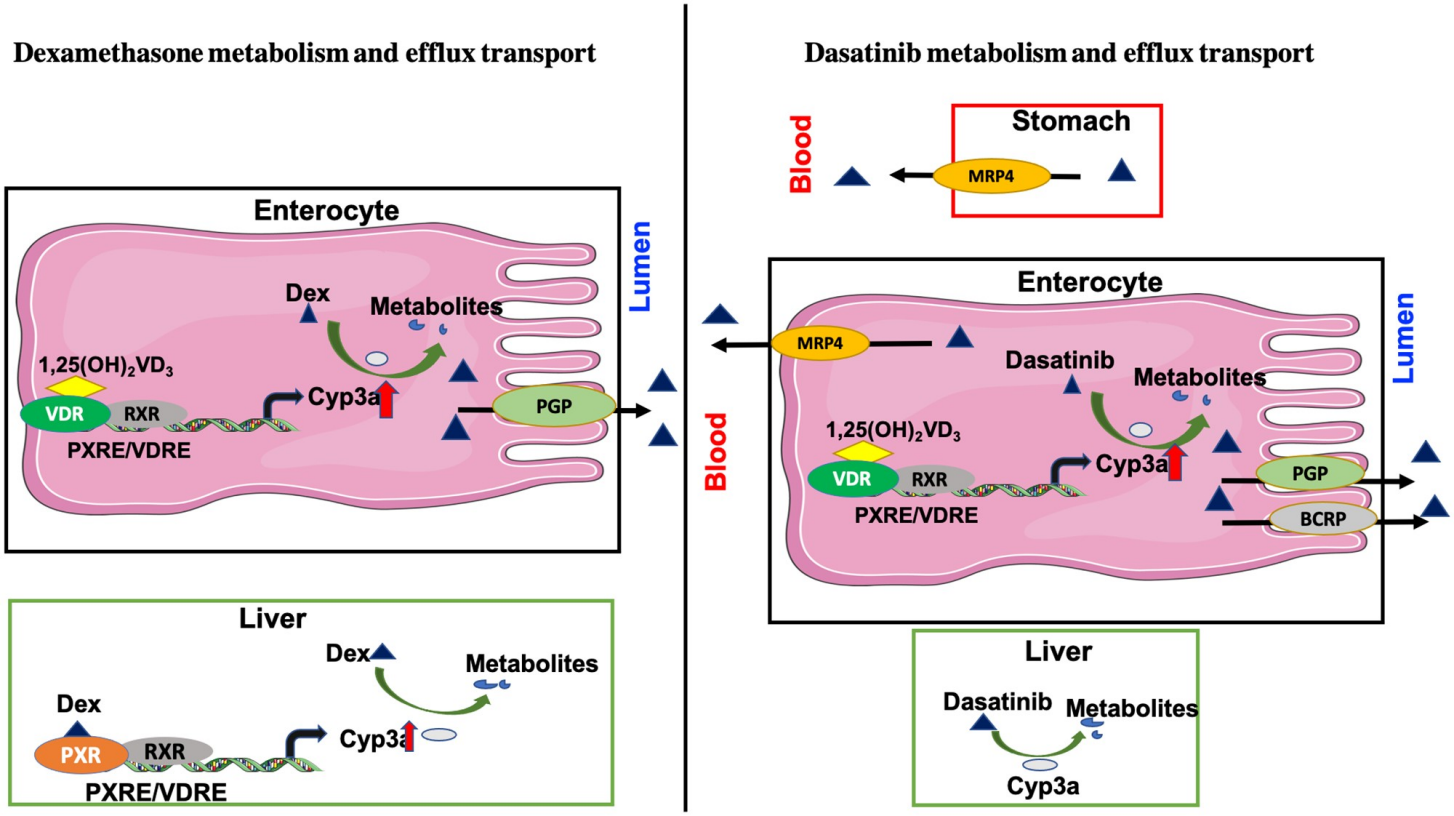
Cartoon depicting dexamethasone and dasatinib metabolism and efflux transport in intestine and liver. Created with Creative Commons Attribution 3.0 Unported license (CC BY 3.0; http://www.servier.com/Powerpoint-image-bank).

While we chose to test the effect of VD_3_ levels on DEX systemic exposure because it is an orally administered anti-leukemic therapy, we would not *a priori* have predicted that intestinal dexamethasone CYP3A-mediated metabolism would be affected by VD_3_ levels for several reasons (Fig 7). First, DEX has a relatively high oral bioavailability in healthy individuals (76% and 81% [30]). Hence, even though DEX can be metabolized by intestinal tissue [31], CYP3A-mediated DEX first-pass metabolism in the gut is probably minimal. Second, DEX is known to be primarily cleared by hepatic pathways [27, 32] undergoing extensive CYP3A4 dependent hepatic metabolism *in vivo* and *in vitro* [33], and there was no effect of VD_3_ status on mouse hepatic Cyp3as consistent with the low levels of VDR in hepatocytes compared to intestine [34]. Hence, it was not clear that vitamin D induced variation in intestinal CYP3A4 expression would impact oral DEX systemic exposure.

We chose to test the effect of VD_3_ levels on dasatinib pharmacokinetics because this CYP3A substrate is given orally throughout therapy for BCR-ABL ALL. We would have predicted that dasatinib oral bioavailability might be affected by VD_3_ levels (Fig 7). Dasatinib is rapidly absorbed from the gastrointestinal tract after oral dosing, with the Tmax reached in nude female nude mice at 2 hrs, in Mdr1a wild-type and KO mice at 0.25 hr, and in monkeys at 0.6 hr [35]. Bioavailability of dasatinib is quite variable with one study showing 45–51% bioavailability [36] and another indicating 17% in mice [35]. Using bile duct cannulated rats, the percentage of unchanged drug in the gastrointestinal tract was 33% suggesting 67% was absorbed. However, the oral bioavailability in these rats was 27%, which means 63% of drug did not reach the systemic circulation, possibly due to pre-systemic metabolism. Moreover, at least 40% of the total drug (or 60% of absorbed drug) might undergo hepatic metabolism. Dasatinib is primarily metabolized by CYP3A4. How much does intestinal CYP3A4 contribute to dasatinib metabolism? On the one hand co-administration of dasatinib with grapefruit juice is contraindicated [37] suggesting there is intestinal CYP3A4 metabolism, although inhibition of Bcrp could also contribute to the dasatinib-grapefruit juice interaction [38]. Moreover, the magnitude of reported interaction of dasatinib with potent CYP3A inducers [39, 40] and inhibitors supports the assumption of first pass at both intestine and liver. However, it should also be considered that CYP3A4-mediated dasatinib metabolism generates CYP3A4 mechanism-based inactivators with a K(I) = 6.3 microM and K(inact) = 0.034 min (-1) [41]. In our study dasatinib was dosed at 10 mg/kg, the calculated Cgut is 1.5 mM (10 mg/kg into a gastric volume of 300–400 μL). Hence, it is possible that intestinal CYP3A4 undergoes mechanism-based inhibition by CYP3A4-generated dasatinib metabolites.

While the current mouse study was primarily designed to evaluate the effect of Vitamin D status on apparent oral Cl of dasatinib, and to explore any effect of sex, a limitation of the study is the sample size of eighteen mice total, balanced for sex, that were allocated to each parallel Vitamin D status group. Although the population Cl estimate was precise, with a relative standard error (RSE) of 6.66%, and the inter-individual variance was well estimated (20% RSE), considering the log-normally distributed variability in Cl, and assuming only an effect of Vitamin D without confounding sex effect, 44 mice in each parallel Vitamin D group would be required to determine a 17% difference in Cl, with an alpha = 0.1 and beta = 0.20. However, even if we had used 44 mice in each group, such small effect sizes are very difficult to determine in preclinical mouse studies, and ultimately are of questionable clinical relevance.

**Is there an intestinal drug efflux transporter-mediated Vitamin D**_**3**_ **interaction with either dexamethasone or dasatinib?** An additional variable affecting oral DEX intestinal absorption could be intestinal P-gp [22]. We first demonstrated that intestinal P-gp is highly inducible not only by drugs [42] but also by vitamin D exposure [7]. Hence, in theory variable VD_3_ levels could influence intestinal P-gp expression and contribute to variable DEX absorption and systemic exposure. Dasatinib is also a substrate of P-gp/Mdr1a and Bcrp that are present at significant levels on the apical membrane of intestinal enterocytes and each has been shown to contribute to limiting dasatinib’s oral bioavailability [38, 43, 44]. Conversely, evidence suggests there was a significantly lower dasatinib AUC in Mrp4 KO vs. WT mice supports Mrp4 enhanced dasatinib gastric absorption [45]. But only Mdr1a has been shown to be induced in enterocytes treated with vitamin D [7]. However, not only did our study find no effect of VD_3_ levels in mice on dasatinib’s AUC, nor any chronic effect on DEX systemic exposure, in fact the expression of Mdr1a mRNA was actually higher in the intestines of VD_3_ deficient vs. sufficient mice, a paradoxical finding that others have also reported [46], but that may contribute to the paradoxical finding that the blood concentration of DEX following a single dose gavage was significantly higher in VD_3_ deficient mice (S1 Fig).

**It is also possible that the paradoxical decrease in intestinal drug efflux transporters for dexamethasone (Pgp, Bcrp) and dasatinib (Pgp) in VD**_**3**_ **sufficient mice (**Fig 1**) negated the increased intestinal Cyp3a in these same mice.** VD_3_ deficient mice had significantly lower intestinal Cyp3a expression, but significantly higher Mdr1a levels. The combined opposing effects of metabolism and efflux transport on DEX and dasatinib in this model may have ultimately erased any individual effect of VD_3_ on their metabolism or transport.

**Fig 8:**
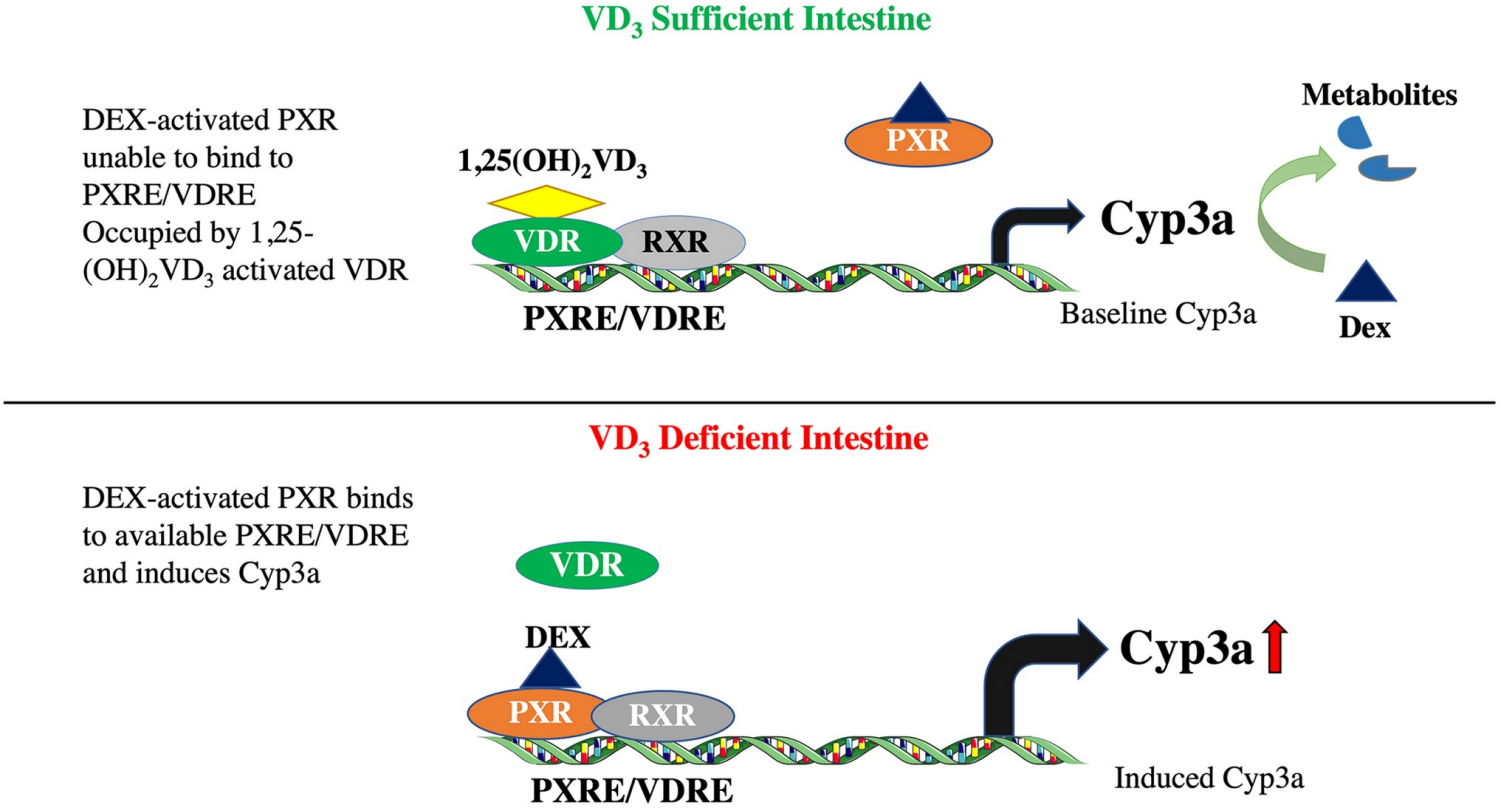
Cartoon depicting hypothesis that dexamethasone-activated PXR can induce Cyp3a in VD_3_ deficient mice, but not in VD_3_ sufficient mice where 1,25(OH)_2_D_3_ activated VDR blocks DEX-PXR induction. Created with Creative Commons Attribution 3.0 Unported license (CC BY 3.0; http://www.servier.com/Powerpoint-image-bank).

**Why was there selective DEX induction of intestinal Cyp3a in VD**_**3**_ **deficient mice, but not sufficient mice (**Fig 4**)?** The inability of DEX to induce Cyp3a in VD_3_ sufficient mice was not because some theoretical maximum intestinal Cyp3a expression level had already been achieved as the threshold cycle (Ct value) for Cyp3a amplification was lower in VD_3_ deficient mice treated with DEX then in vitamin D sufficient mice with or without DEX. This means the relative expression level of Cyp3a in the DEX induced VD_3_ deficient mice was higher than the Cyp3a level in sufficient mice. One hypothetical explanation is that there is competition of VDR and PXR at the VDRE/PXRE binding sites in the Cyp3a promoter (Fig 8). VDR/NR1I1 and PXR/NR1I2, both members of the NR1I family of nuclear hormone receptors, share common DNA binding elements [47]. We previously showed that VDR-RXR and PXR-RXR heterodimers bind to the identical DNA elements in the Cyp3a promoter [7]. Moreover, there is precedence for VDR-RXR and PXR-RXR heterodimers competing at common binding elements because PXR bound by the potent ligand rifampin was previously shown to inhibit VD_3_-ligand activated VDR binding to the CYP24 promoter in intestinal cells [48]. Results from this study suggest that, in sufficient mice, VD_3_ activated VDR-RXR bound to the Cyp3a VDRE/PXRE is preventing DEX (a relatively weak PXR agonist (Kd~30 μM))-activated PXR-RXR from displacing VDR from the VDRE/PXRE binding site (Fig 8). Conversely, in VD_3_ deficient mice the VDRE/PXRE is unoccupied by VDR because the VD_3_ ligand level is insufficient to ligand activate DNA binding, so the DEX-activated PXR-RXR can readily bind to and activate the Cyp3a promoter (Fig 8).

**Fig 9:**
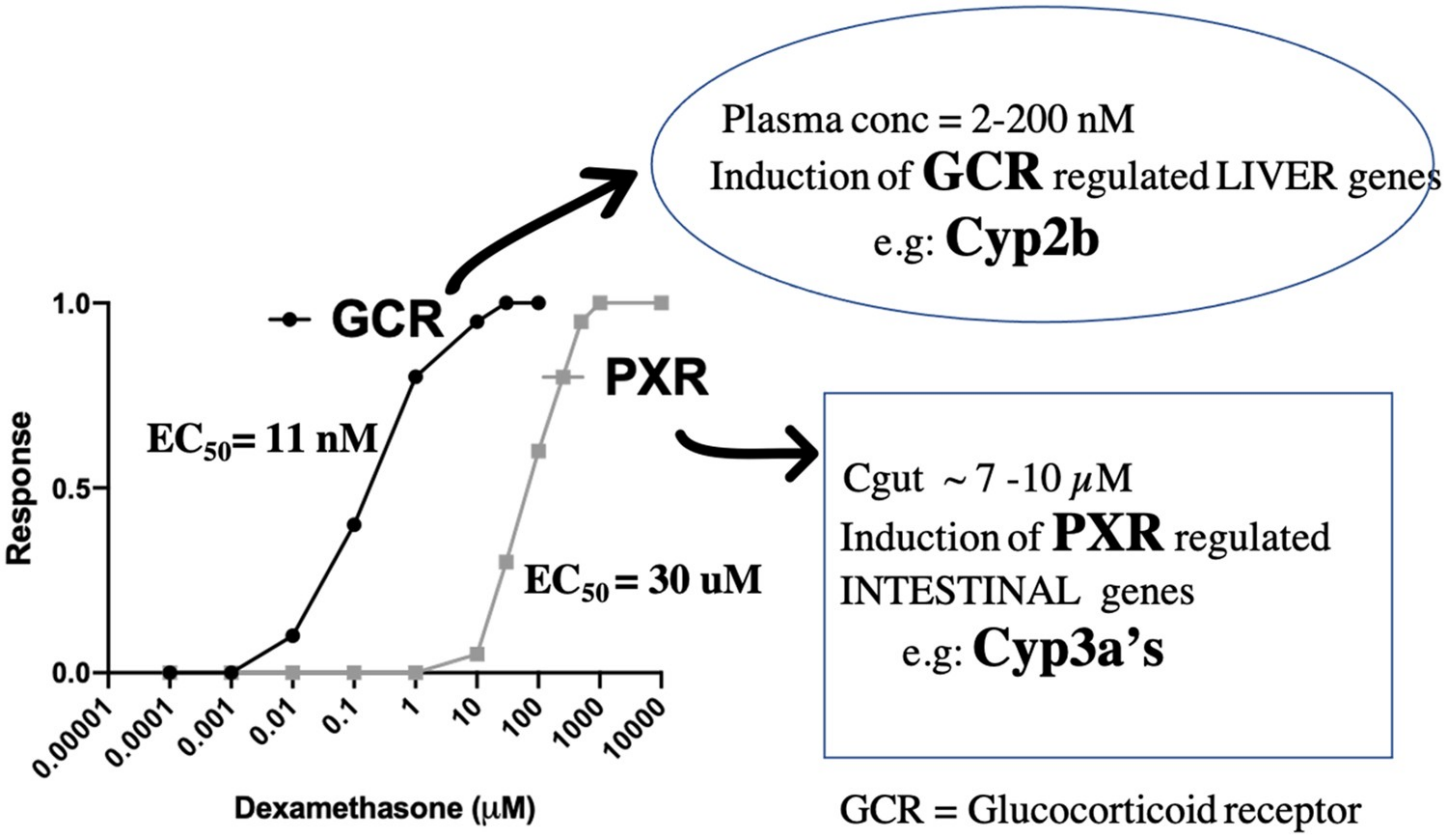
Differences in plasma and gut dexamethasone concentrations following the oral discontinuous DEX regimen leads to tissue specific activation of GCR and PXR. The DEX plasma concentration range was determined from the discontinuous DEX PK study. The DEX Cgut from drinking water is ~7.6–10.1 μM assuming mice are drinking the typical 200–400 μl/hr. *Kliewer SA*, *et al*. *Cell*. *1998*.*PMID*: *9489701 DMD 31*:*548–558*, *2003* *J Biol Chem*. *2002 Jan 11; PMID*: *11682470*.

**Tissue-specific DEX induction of Cyp3a in intestine but not liver, and DEX induction of Cyp2b in liver, suggests compartment specific induction of these Cyps by PXR and the glucocorticoid receptor (GCR) (**Fig 9**).** The dose response curves for glucocorticoid activation of GCR vs. PXR are strikingly different (Fig 9). GCR is ligand activated at nanomolar concentrations of endogenous glucocorticoids and this makes physiological sense as these steroids need to be maintained within a strict homeostatic range. In contrast, PXR activation by glucocorticoids begins in the μM range [49], a level only achieved *in vivo* with physiological stress [50] and the ED_50_ for PXR activation by glucocorticoids is approximately 30 μM [49]. If PXR were activated by glucocorticoids at nanomolar concentrations this would profoundly perturb endogenous glucocorticoid homeostasis since PXR induces clearance of steroids through induction of Cyps and transporters. In contrast, Cyp2b is transcriptionally activated by the glucocorticoid receptor and we previously showed GCR is required for DEX induction of Cyp2b in mice [51]. Conversely, glucocorticoid induction of Cyp3a occurs via PXR with no participation of, or requirement for, GCR [51]. The observed systemic concentrations of DEX (2.6–201.5 nM) in mice on the discontinuous regimen were in the nanomolar range that would activate GCR, but not PXR, in liver. Accordingly, only Cyp2b was induced by DEX-activated GCR in liver (Fig 4). However, the gut concentration of orally administered DEX (Cgut is ~7.6–10.1 μM) would readily activate intestinal PXR and induce Cyp3a. Importantly, these results are directly relevant to the pediatric ALL patients treated with DEX as the plasma concentrations achieved with the oral discontinuous regimen in mice are similar to those achieved in patients treated with conventional DEX therapy [21].

We recently reported in a murine BCR-ABL ALL model that VD_3_ sufficient mice died earlier than VD_3_ deficient mice [16]. Given our finding that VD_3_ deficient mice had higher systemic DEX exposure compared to sufficient mice during the first days of discontinuous DEX treatment we might have expected DEX treatment to negate this VD_3_ deficient survival advantage. However, DEX treatment was unable to reverse the survival advantage of VD_3_ deficient over sufficient mice with ALL. It remains to be determined whether the effects of VD_3_ on survival of mice from BCR-ABL ALL translate to humans.

The 5-year event free survival rates for pediatric ALL have almost reached 90% [52]. However, it is always important to consider whether a therapeutic intervention–e.g., vitamin D3 to treat vitamin D deficiency, might cause an unexpected negative side-effect–in this case an unfavorable CYP3A vitamin D3-mediated drug interaction. Our results in mice suggest that vitamin D supplementation to vitamin D insufficient/deficient patients will not affect systemic exposures of DEX or dasatinib in a clinically impactful manner. Our results do suggest the intriguing possibility that whether a patient is VD_3_ sufficient vs. deficient may have an impact on the magnitude of CYP3A-mediated drug interaction with CYP3A inducers (including DEX) for substrates that undergo significant first pass intestinal metabolism (e.g., atorvastatin) [11].

## Supporting information

S1 Text Report on plasma pharmacokinetics of oral dasatinib in C57BL/6 mice grouped by vitamin D status, sex, and age from the St Jude Children’s Research Hospital preclinical PK shared resource. (PDF)

S1 Fig Dexamethasone plasma pharmacokinetics in male and female VD_3_ sufficient and deficient mice, aged 14 weeks, after a single oral gavage of 2 mg/kg dexamethasone phosphate. Observed and model predicted dexamethasone plasma concentration vs. time (Ct) profiles by sex (panels) and VD_3_ status. The black solid (VD_3_ Sufficient) and gray dashed (VD3 Deficient) lines indicate the population model predicted median concentrations, whereas the shaded areas represent the model’s 90% prediction intervals (90% PI) by VD_3_ status. There were statistically significant differences in dexamethasone PK between sexes, with males having approximately 25% lower AUC values (p<0.05). VD_3_ deficiency also resulted in an approximately 25% lower AUC value across both sexes (p<0.05).
(TIF)

S1 Table Gene expression (qPCR) data from VD3 sufficient and deficient C57BL/6 mice duodenum. (Data supporting Fig 1).
(XLSX)

S2 Table Gene expression (qPCR) data from VD3 sufficient and deficient hCYP3A4 12-week-old mice duodenum. (Data supporting Fig 1).
(XLSX)

S3 Table Gene expression (qPCR) data from VD3 sufficient and deficient hCYP3A4 12-week-old mice liver and kidney. (Data supporting Fig 2).
(XLSX)

S4 Table Discontinuous DEX PK raw data from repeated study. (Data supporting Fig 3).
(XLSX)

S5 Table DEX oral gavage PK raw data. (Data supporting S1 Fig).
(XLSX)

S6 Table Gene expression (qPCR) data from duodenums of C57BL/6 mice before and after DEX treatment. (Data supporting Fig 4A and 4B).
(XLSX)

S7 Table Gene expression (qPCR) data from livers of C57BL/6 mice before and after DEX treatment. (Data supporting Fig 4C and 4D).
(XLSX)

S8 Table Dasatinib oral gavage PK raw data. (Data supporting Fig 5).
(XLSX)

S9 Table Raw data from survival study in VD_3_ sufficient vs. deficient male mice from BCR-ABL ALL when treated with dexamethasone. (Data supporting Fig 6).
(XLSX)

S10 Table Proportion of VD_3_ sufficient vs. deficient mice surviving from BCR-ABL ALL when treated with dexamethasone. (XLSX)
