# Supplementary material for: Vitamin D levels do not cause vitamin-drug interactions with dexamethasone or dasatinib in mice
Source: PLoS One. 2021 Oct 20;16(10):e0258579. doi: 10.1371/journal.pone.0258579 (PMC8528301; doi:10.1371/journal.pone.0258579)
Supplement: S1 Text — (PDF) [file pone.0258579.s001.pdf]

St. Jude Children's Research Hospital
ALSAC • Danny Thomas, Founder
Finding cures. Saving children.

PRECLINICAL PHARMACOKINETIC REPORT

Pharmaceutical Sciences Department

P-PKSR Study 126148-1319026

STUDY TITLE:

PLASMA PHARMACOKINETICS OF ORAL DASATINIB IN C57BL/6 MICE, GROUPED BY VITAMIN D STATUS, SEX, AND AGE

**SHORT TITLE:**	**Dasatinib VitD DDI**	
**TEST ARTICLE:**	Dasatinib (free base)	
**SECTION:**	Nonclinical Pharmacokinetics (Non-GLP)	
**PRINCIPAL INVESTIGATOR(S)**	Schuetz, Erin <Erin.Schuetz@STJUDE.ORG>	
**SJCRH SRM2 O/R:**	126148-1319026	Preclinical Pharmacokinetic Shared Resource
	139813-1465178	Preclinical Pharmacokinetic Shared Resource
**REFERENCE STUDY NUMBERS:**	NA	NA
**IN VIVO SCIENTIST(S)**	Annu, Kavya <Kavya.Annu@STJUDE.ORG><br>Charlton, Gregory <Gregory.Charlton@STJUDE.ORG>	
**BIOANALYTICAL SCIENTIST:**	Caufield, William <William.Caufield@STJUDE.ORG>	
**REPORT AUTHOR(S):**	Freeman, Burgess <Burgess.Freeman@STJUDE.ORG>	
**REPORT FORMAT:**	Manuscript Supplementary Materials	
**REPORT STATUS:**	FINAL	
**DATE:**	2018-08-27	

FOR P-PKSR APPROVED USE AND DISTRIBUTION

Quality Statement

This non-GLP study was conducted using sound scientific principles and established techniques in accordance with the relevant guidelines and standard operating procedures (SOPs) of the Preclinical Pharmacokinetic Shared Resource and St. Jude Children's Research Hospital, Memphis, TN, USA. This report accurately reflects the data obtained during the course of this study.

These results represent part of an early phase preclinical pharmacology program. This study has been conducted to provide preliminary insights into the pharmacokinetic (PK) properties of the compound(s) in the indicated preclinical model(s). This study and its results are not intended to provide a comprehensive PK evaluation of the compound(s). The applied bioanalytical method was validated/qualified to support this specific study and discovery-style sample analyses.

Substantial study-to-study and inter-animal variability in preclinical PK exists. Such variability depends upon the in vivo scientists' experience, variations in compound purity and formulation, animal strains, sex and age, and other situational fixed effects (i.e. husbandry conditions, chow constituents, presence or absence of disease, concomitant drugs). As such, the actual PK, plasma or tissue compound concentrations, or equivalent dose in other studies or preclinical models may vary significantly from that reported herein.

Signatures (Nonregulated Report)

Approved By:

Digitally signed by Freeman, Burgess
DN: cn=Freeman, Burgess, o=St. Jude Children's Research Hospital, ou=Preclincal Pharmacokinetic Shared Resource, email=burgess.freeman@stjude.org, c=US
Date: 2018.08.27 15:16:33 -05'00'

Burgess B. Freeman III, PharmD
Director
Preclinical Pharmacokinetic Shared Resource
St. Jude Children's Research Hospital

Date

1.0 OBJECTIVES

- To evaluate the plasma pharmacokinetics (PK) of dasatinib after two separate single oral gavage doses of solution, separated by a washout period, in normal C57BL/6 mice
- To assess the effects of Vitamin D dietary status (VD3 Suf vs VD3 Def), sex (M vs F) and age (8wk vs 10wk) on the plasma PK of oral dasatinib using a nonlinear mixed effects (NLME) PK modeling approach

2.0 MATERIALS AND METHODS

2.1 In Vivo Pharmacokinetic (PK) Study

The total plasma PK of dasatinib in C57BL/6 mice (The Jackson Laboratory) was assessed after single oral gavages of 10 mg/kg of dasatinib free base equivalents. Dasatinib (St. Jude Compound Management, SJ000518976-9, LC Labs, D-3307, Lot BDS-106, purity >99%) was dissolved in 80 mMol/L sodium citrate buffer pH 3.1 for a final dasatinib concentration of 1 mg/mL for a 10 mL/kg gavage volume. Mice were grouped by Vitamin D dietary sufficiency status and sex, and studied at 8 and 10 weeks of age. Survival saphenous and facial vein bleeding of mice was conducted using IACUC-approved methods at 0.125, 0.25, 0.5, 1, 2, 4, 8, 16 and 24 hr. post-dose, with 3 mice per time point. Each mouse was sampled 3 times on the 2 separate occasions of dasatinib dosing. Blood was collected into a Sarstedt Minivette® POCT 50 µl K3 EDTA capillary device, dispensed into a microtube and vortexed to mix the anticoagulant. The tubes were then immediately centrifuged to plasma, and stored on dry ice for the remainder of the study. At the end of the in vivo procedures, plasma samples were transferred from dry ice and placed at -80 °C until analysis.

2.2 Bioanalysis

Total plasma dasatinib concentrations were assessed using a sensitive and specific liquid chromatography, tandem mass spectrometry assay. Dasatinib (St. Jude Compound Management, SJ000518976-9, Purity >99%) stock solutions were prepared in acetonitrile and used to spike matrix calibrators and quality controls. Protein precipitation was performed using a 1: 4 ratio of plasma to 15 ng/mL erlotinib HCl (St. Jude Compound Management, S00004053, Inventory ID: SJCH0042996, Purity >95%) in methanol as an internal standard. A 3 µL aliquot of the extracted supernatant was injected onto a Shimadzu LC-20ADXR high performance liquid chromatography system via a LEAP CTC PAL autosampler. The LC separation was performed using a Phenomenex Kinetex 2.6 µm EVO C18 (100 Å, 50 x 2.1 mm) maintained at 50 °C with gradient elution at a flow rate of 0.5 mL/min. The binary mobile phase consisted of water: acetonitrile: 200 mM ammonium acetate in H_2O pH = 6.0 (9:1:1 v/v) in reservoir A and acetonitrile: H_2O: 200 mM ammonium acetate in H_2O pH = 6.0 (9:1:1 v/v) in reservoir B. The mobile phase gradient began with a linear increase to 100% B in 4.0 minutes. The column was then rinsed for 2.0 minutes at 100% B and then equilibrated at the initial conditions for two minutes for a total run time of eight minutes. Under these conditions, the analyte and IS eluted at 1.81 and 2.21 minutes, respectively.

Analyte and IS were detected with tandem mass spectrometry using a SCIEX API 5500 Q-TRAP in the positive ESI mode with monitoring of the following mass transitions: dasatinib 488.20 -> 401.00, erlotinib HCl 394.20 -> 250.10.

The experimental bioanalytical runs were all found to be acceptable for the purpose of a singlicate non-GLP, preclinical PK assessment. A linear model ($1/X^2$ weighting) fit the calibrators across the 1.00 to 500 ng/mL range, with a correlation coefficient (R) of ≥0.9958. The lower limit of quantitation (LLOQ), defined as a peak area signal-to-noise ratio of 5 or greater verses a matrix blank with IS, was 1.00 ng/mL. The intra-run precision and accuracy was < 11.6% CV and 90.6% to 107%, respectively.

2.3 Pharmacokinetic Analysis

Plasma concentration-time (Ct) data in ng/mL for dasatinib were grouped by individual mouse, Vitamin D status, sex, and age, and were analyzed using nonlinear mixed effect (NLME) modeling as implemented in Monolix version 2018R1 (Lixoft SAS, Antony, France). Briefly, parameters and the Fisher Information Matrix (FIM) were estimated using the stochastic approximation expectation maximization (SAEM) algorithm, and the final log-likelihood estimated with importance sampling, all using the default Monolix initial settings, except that 1000 iterations were permitted for estimation of FIM using stochastic approximation. A variety of models were fit to the dasatinib Ct data, parameterized using apparent clearances, volumes of distribution, and absorption rate constant as needed. These models were assessed for goodness of fit using the -2 log likelihood (-2LL) value, Akaike and Bayesian Information Criterion (AIC, BIC), visual predictive checks, plots of model individual and population predicted vs. observed data, residual plots, and the standard error of parameter estimates. A log-normal inter-individual and inter-occasion parameter distribution was assumed on selected supported parameters, with both on- and off-diagonal elements of parameter covariance matrices tested. Additive and/or proportional error models were tested and implemented as supported. Beal's M3 method was used to handle any data that were below the LLOQ or above the upper limit of the assay range [1]. The grouping levels were tested as categorical covariates on supported PK parameters, primarily the apparent oral clearance (Cl). A covariate effect was considered significant if its addition reduced the -2LL by at least 3.84 units ($P < 0.05$, based on the χ^2 test for the difference in the -2LL between two hierarchical models that differ by 1 degree of freedom). Additionally, Wald test P values were outputted for each covariate effect by the Monolix software.

3.0 RESULTS AND DISCUSSION

The oral dasatinib plasma PK in the studied mice was best described using a linear, two-compartment, zero order absorption model, with proportional residual error. Inter-individual variability upon apparent oral systemic clearance (Cl) and apparent volume of distribution of the central compartment (V1) was supported, improved the model fit and performance, as did the off-diagonal correlation between these two parameters. This was defined as the Base model. Inter-occasion variability on either Cl or V1 was not supported, resulted in model instability (failure of FIM convergence via SAEM) and a poorer model fit. This prevented formal testing age as a covariate, but implies that dasatinib PK is not significantly different between 8 and 10 week old mice.

Vitamin D sufficiency and sex were then tested alone and combined as covariates on Cl, and were also found to be insignificant at the predefined $p<0.05$ threshold level. Overall, the Base model without any covariates performed the best, and suggested no influence of Vitamin D status, sex, or age on oral dasatinib PK in mice. **Table 3.1** presents the results from the Base and Final models and **Figure 3.1** shows the population predicted dasatinib plasma concentration-time profiles, the 90% prediction interval, and the observed concentrations for the Final model, showing the lack of appreciable and statistical differences in plasma exposure vs Vitamin D status. Model goodness of fit plots, individual mouse post-hoc fits by occasion, and listings of Ct results are presented in **Appendix 5.1-5.9**.

Table 3.1. NLME PK Modeling Results

		Base Model		Final Model		
**Parameter**	**Units**	**Est**	**%RSE**	**Est**	**%RSE**	**P value**
Tk0_pop	Hr	1.29	16.4	1.33	19.6	
Cl_pop	L/hr/kg	6.73	6.66	7.12	8.82	
beta_Cl_GROUP_VD3_Suf	-			*-0.00958*	*834*	*0.905*
beta_Cl_SEX_M	-			*-0.0874*	*104*	*0.337*
V1_pop	L/kg	12.7	20.8	12.2	25.2	
Q_pop	L/hr/kg	1.17	30.5	1.26	28.4	
V2_pop	L/kg	11.1	26.9	11.2	32.8	
omega_Cl	%CV	0.294	20	0.287	26.7	
omega_V1	%CV	0.465	14.7	0.477	15.4	
corr1_V1_Cl	-	0.762	16.1	0.746	24.3	
b	%	0.433	6.74	0.433	6.66	
-2LL	-	1959.02		1959.99		
AIC	-	1981.02		1977.99		
BIC	-	2006.06		1998.48		

Figure 3.1. Observed and Final Model Predicted (90% Prediction Interval) Dasatinib Ct Profile in Mice Split by Group: Vitamin D Sufficient (VD3 Suf) and Vitamin D Deficient (VD3 Def)

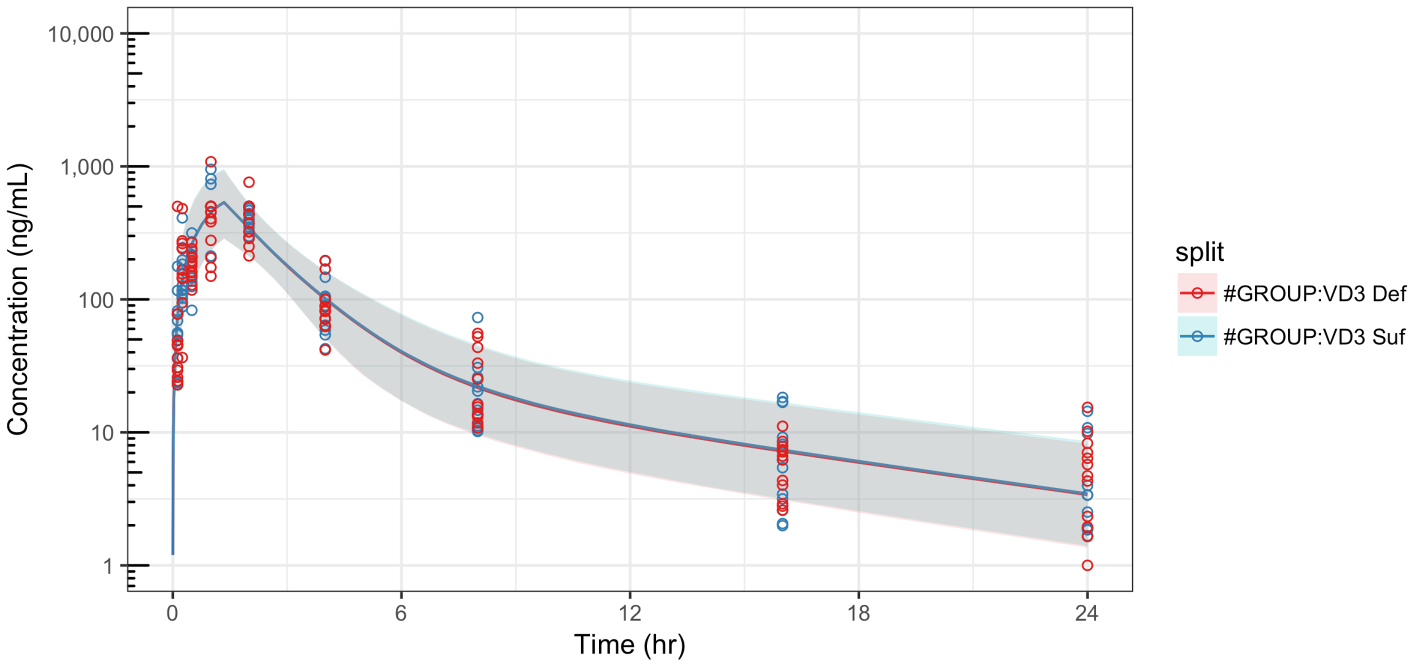

Lindh et al. retrospectively investigated the putative effects of Vitamin D status upon immunosuppressant concentrations extracted from clinical therapeutic drug monitoring (TDM) databases in Sweden [2]. Concentrations for the CYP3A4 substrates tacrolimus and sirolimus showed statistically significant associations with seasons, with apparent oral clearance likely higher in summer months, when Vitamin D

is adequately converted in vivo. Despite the statistical significance, the magnitude of the effect was small – ranging from 4.9% to 17.2%. The authors conceded that while the clinical significance of this effect is unclear, it's likely to be limited given the small effect size. It is unclear what the inter- and intra-assay precision and accuracy values were for these TDM assays, but it's assumed they were at least within standard values of ≤15%. Through the sheer number of samples, the investigators achieved adequate power to detect such small, and likely clinically irrelevant, effects. For reference, these differences are within the FDA guidance for therapeutic bioequivalence by plasma AUCs or clearance values, i.e. ± 20%.

The current mouse study was primarily designed to evaluate the effect of Vitamin D status on apparent oral Cl of dasatinib, and to explore any effect of sex. Eighteen mice total, balanced for sex, were allocated to each parallel Vitamin D status group. The population Cl estimate was precise, with a RSE of 6.66%, and the inter-individual variance was well estimated (20% RSE). Considering the log-normally distributed variability in Cl, and assuming only an effect of Vitamin D without confounding sex effect, 44 mice in each parallel Vitamin D group would be required to determine a 17% difference in Cl, with an alpha = 0.1 and beta = 0.20 [3]. Such small effect sizes are very difficult to determine in preclinical mouse studies, and are of questionable relevance.

4.0 REFERENCES

1. Beal SL. Ways to fit a PK model with some data below the quantification limit. J Pharmacokinet Pharmacodyn. 2001 Oct;28(5):481–504.

2. Lindh JD, Andersson ML, Eliasson E, Björkhem-Bergman L. Seasonal Variation in Blood Drug Concentrations and a Potential Relationship to Vitamin D. Drug Metab Dispos. 2011 May 1;39(5):933–7.

3. Champely S, Ekstrom C, Dalgaard P, Gill J, Weibelzahl S, Anandkumar A, Ford C, Volcic R, Rosario HD. pwr: Basic Functions for Power Analysis [Internet]. 2018 [cited 2018 Aug 27]. Available from: https://CRAN.R-project.org/package=pwr

5.0 APPENDICES

Appendix 5.1. Goodness of Fit Plots for Base Model

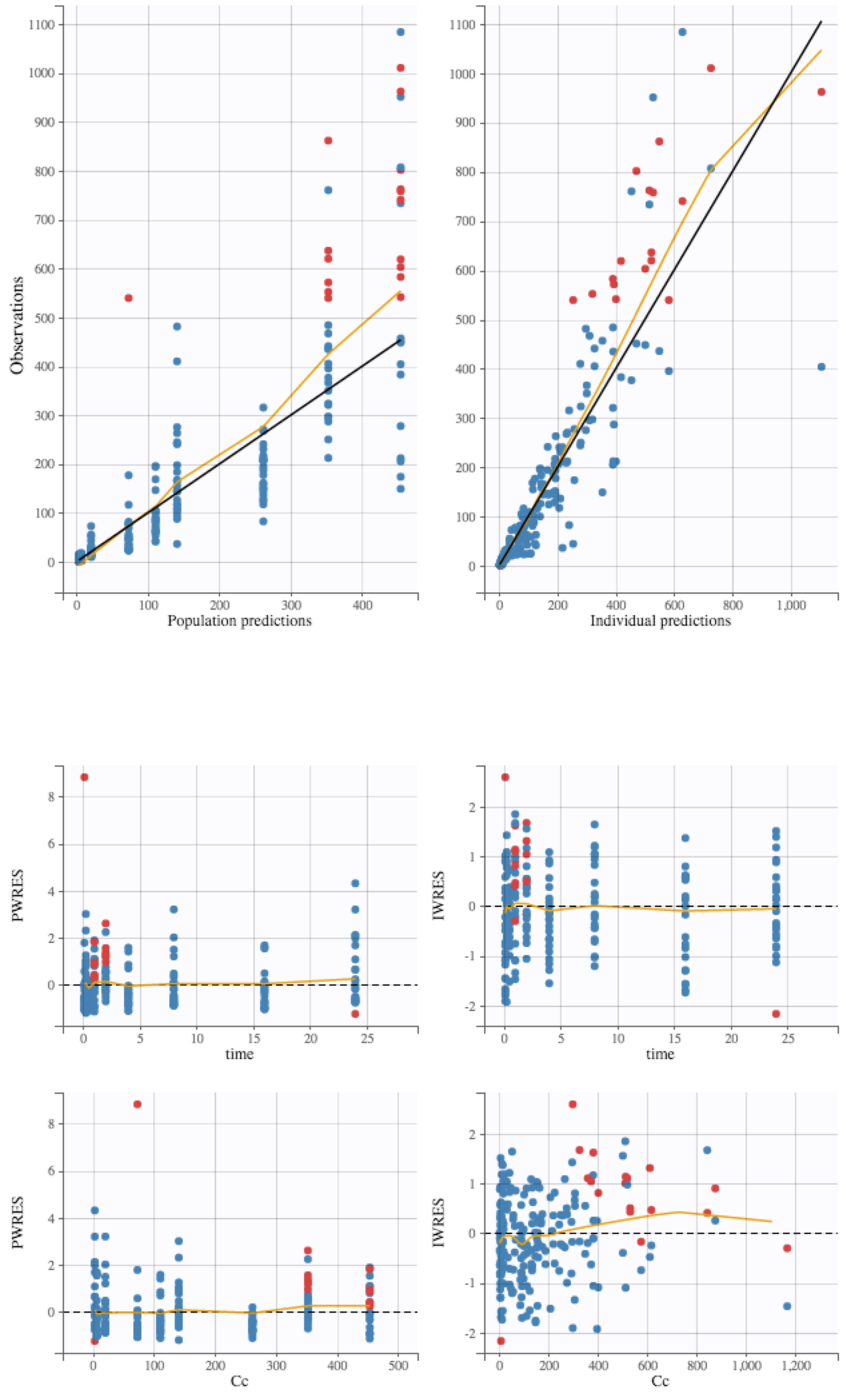

Appendix 5.2. Goodness of Fit Plots for Final Model

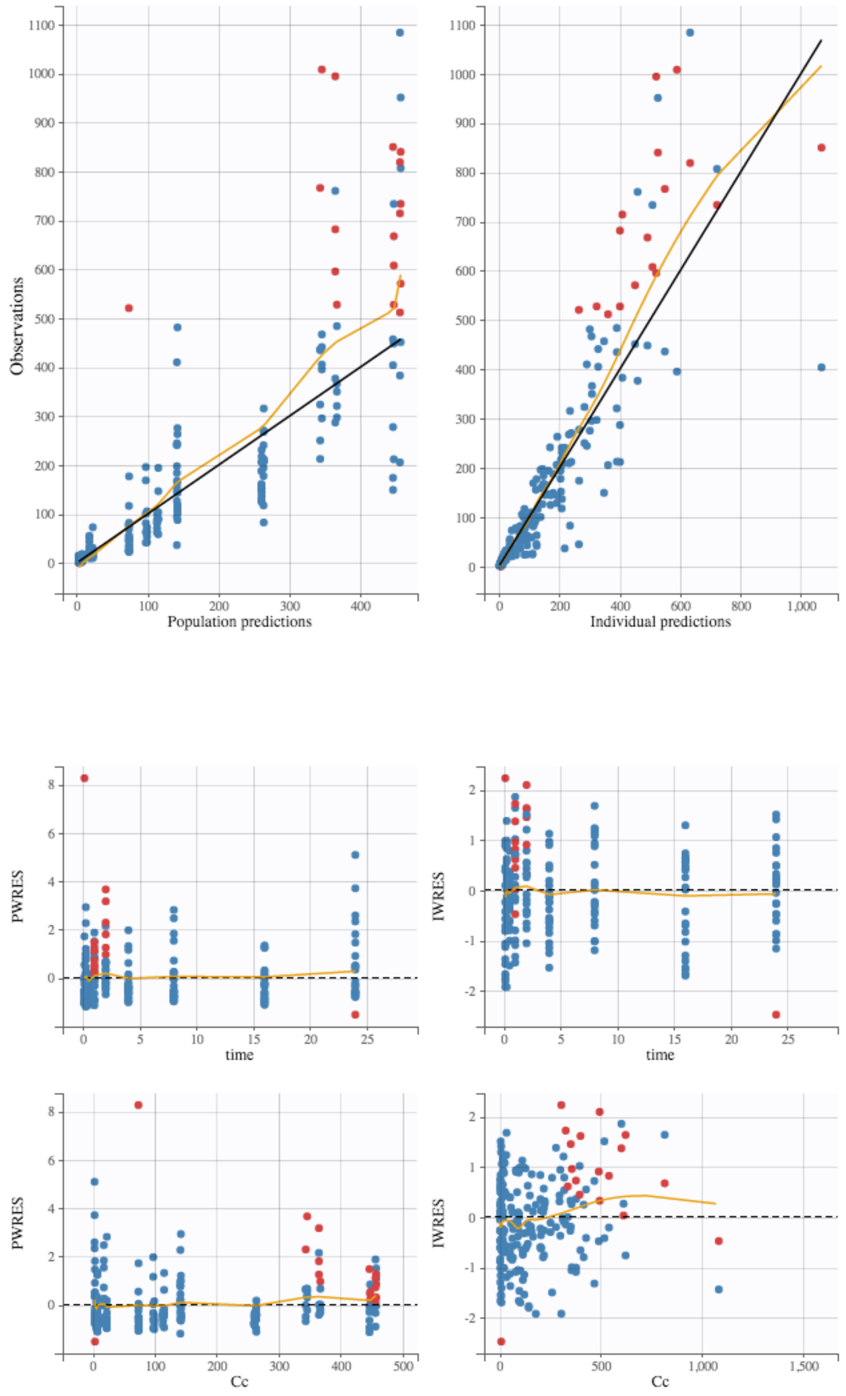

Appendix 5.3. Final Model Post Hoc Individual Fits by Mouse ID and Occasion (M1-M6)

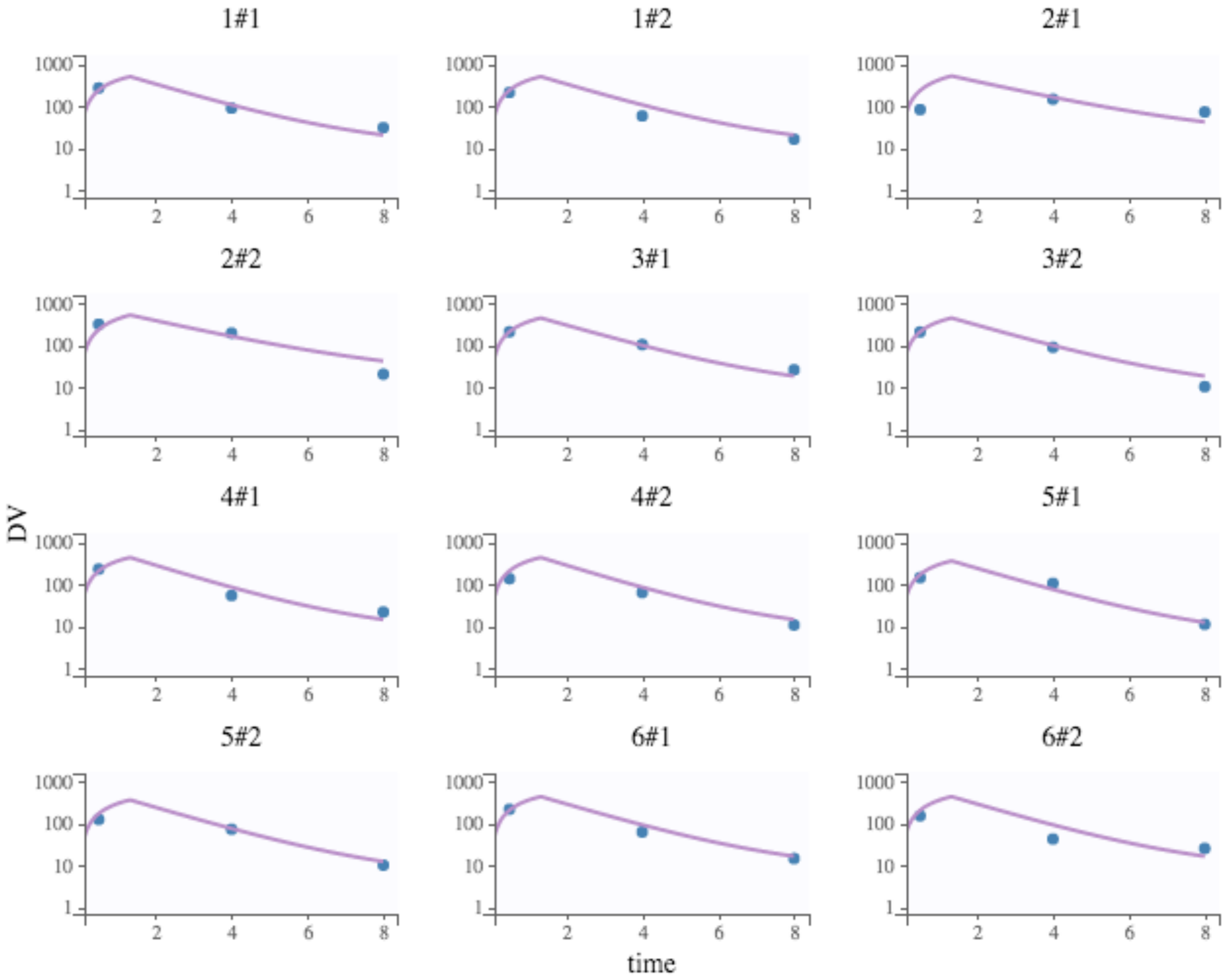

Appendix 5.4. Final Model Post Hoc Individual Fits by Mouse ID and Occasion (M7-M12)

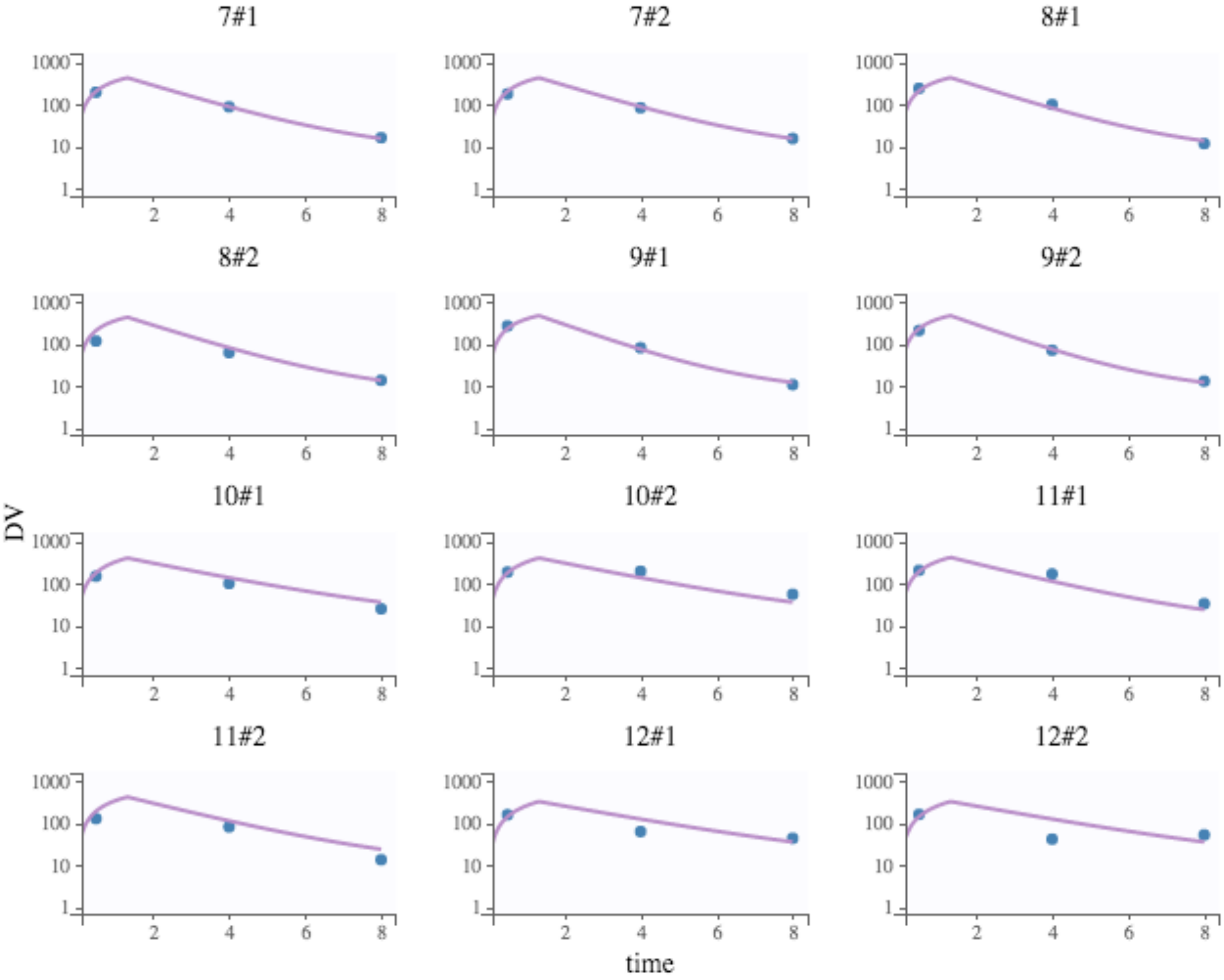

Appendix 5.5. Final Model Post Hoc Individual Fits by Mouse ID and Occasion (M13-M18)

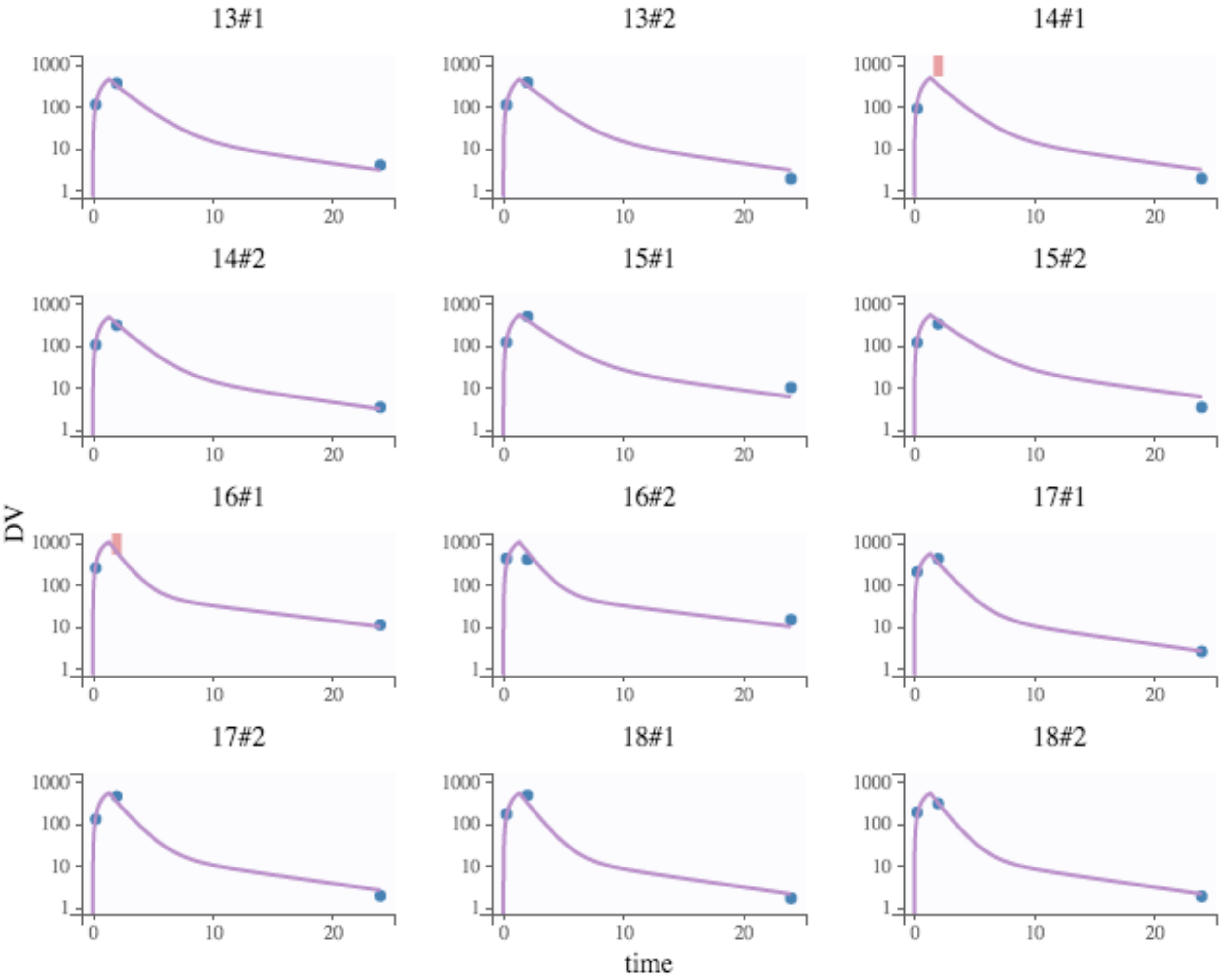

Appendix 5.6 Final Model Post Hoc Individual Fits by Mouse ID and Occasion (M19-M24)

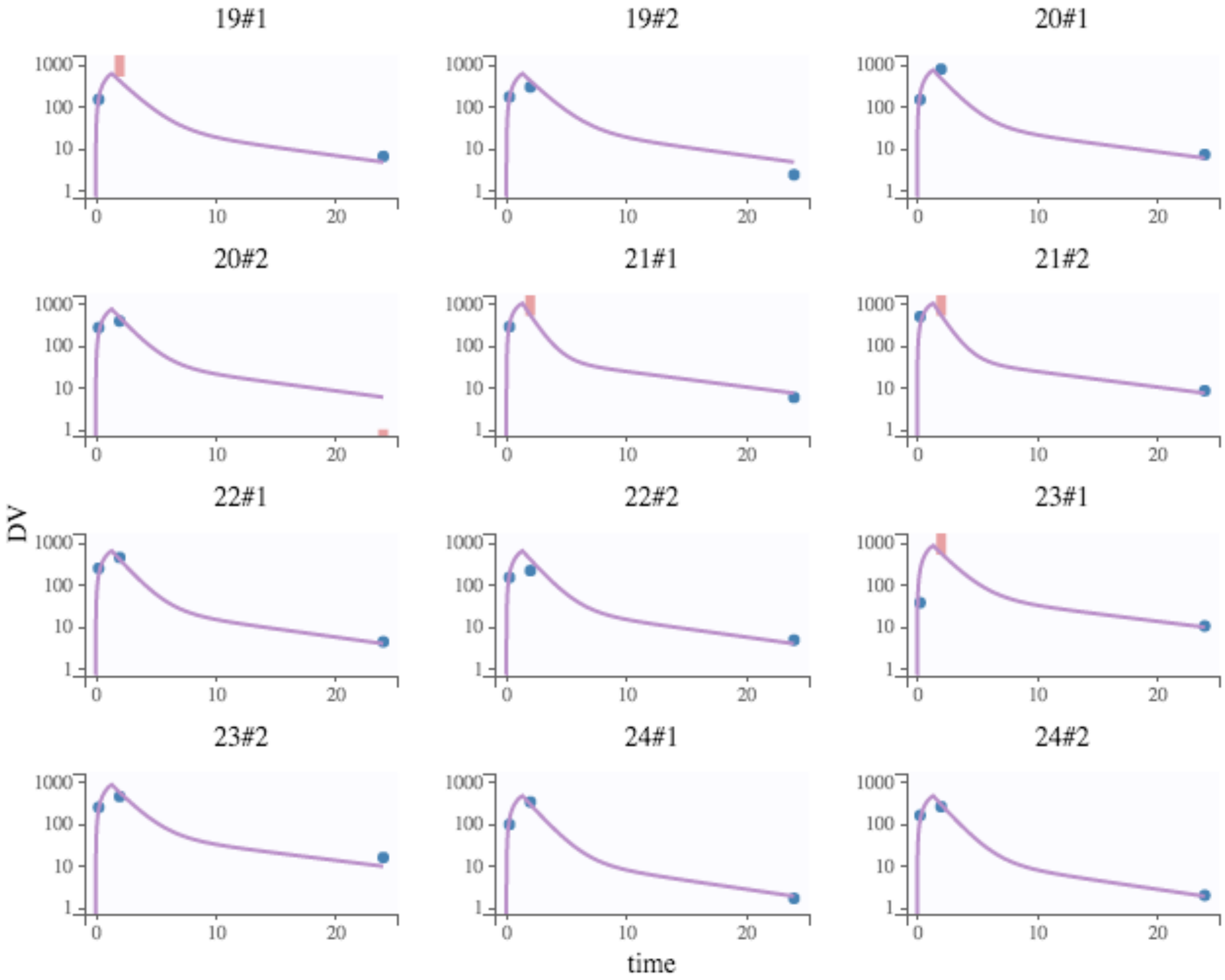

Appendix 5.7. Final Model Post Hoc Individual Fits by Mouse ID and Occasion (M25-M30)

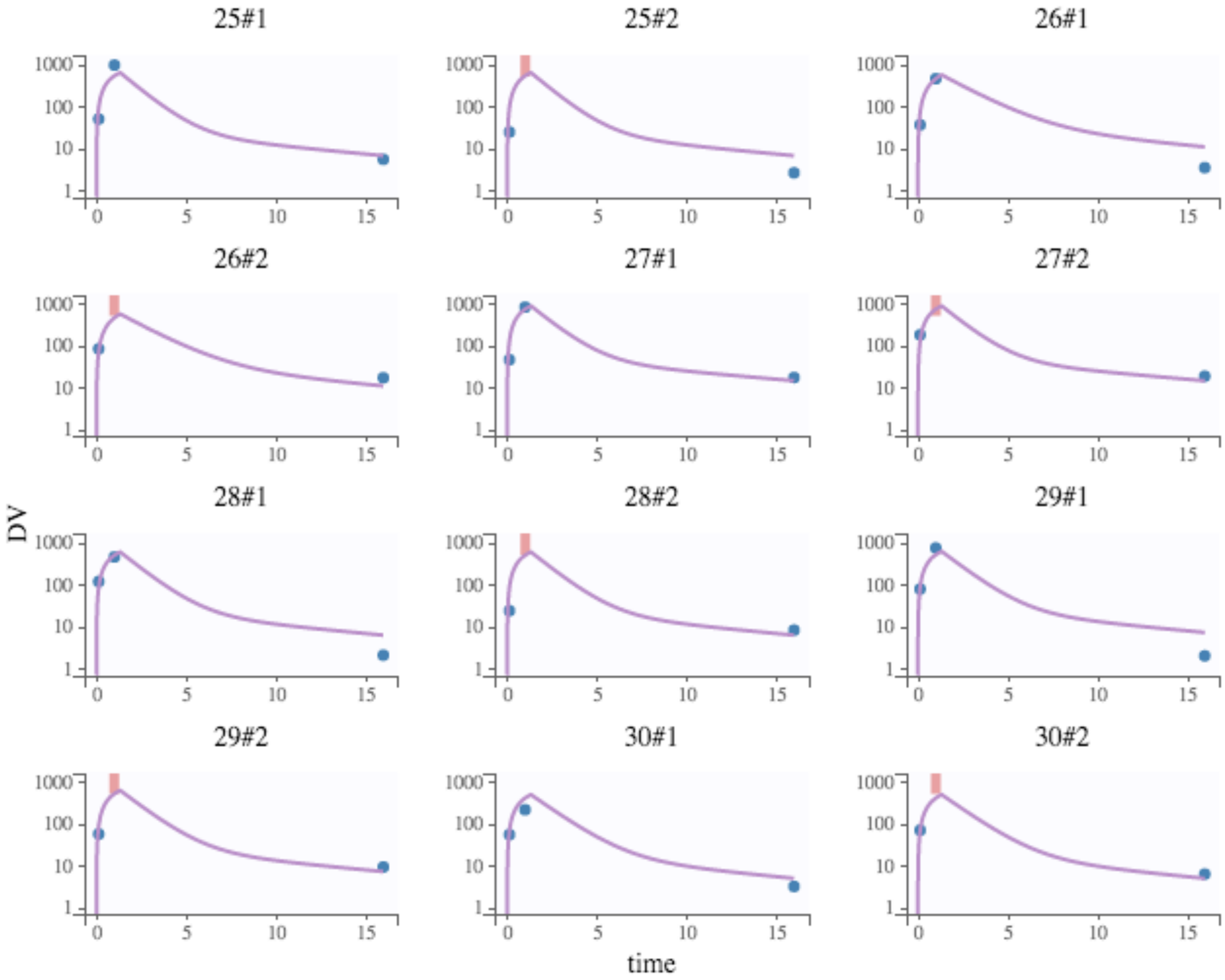

Appendix 5.8. Final Model Post Hoc Individual Fits by Mouse ID and Occasion (M31-M36)

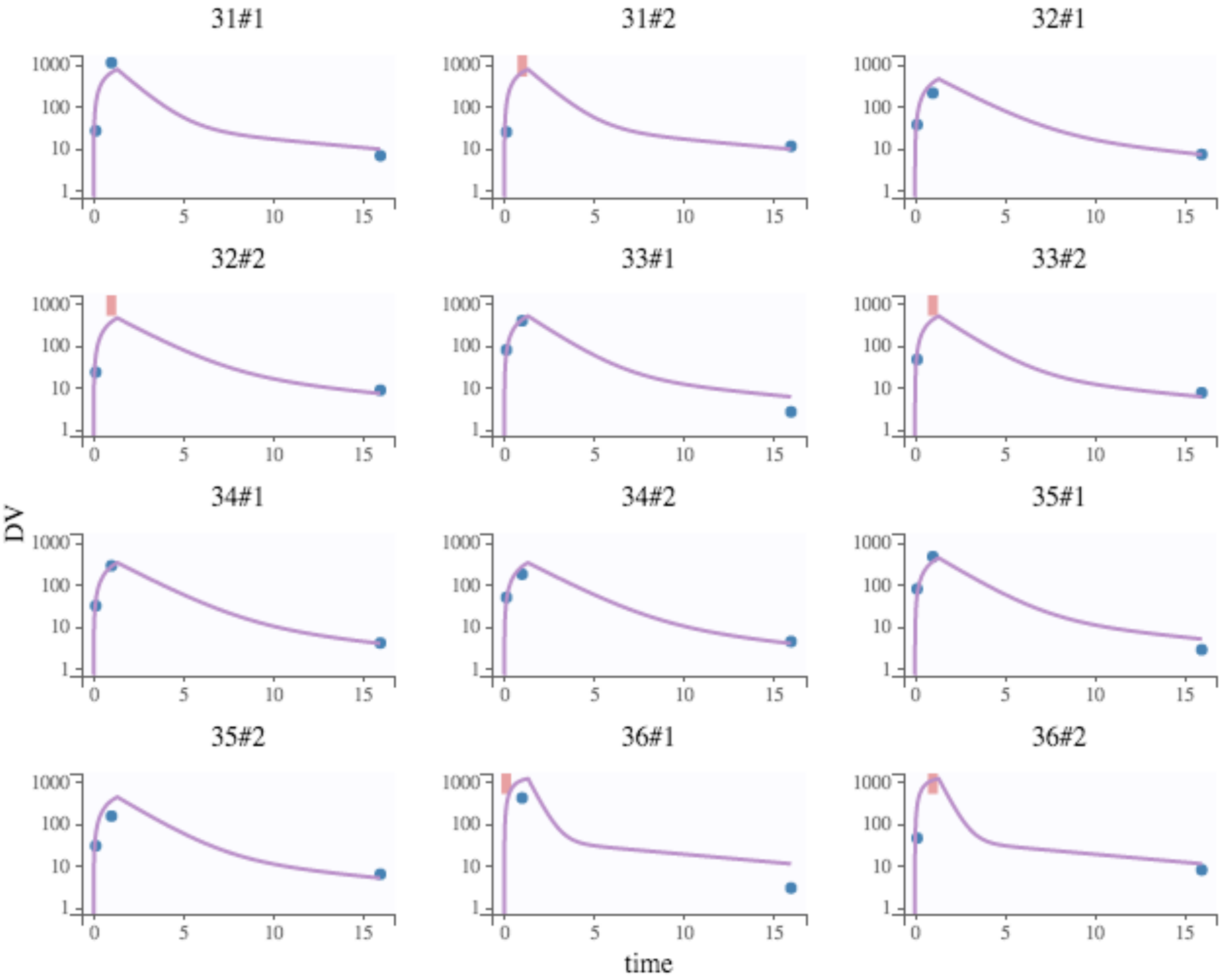

Appendix 5.9. Listing of Dasatinib Ct (DV) Results in Monolix Data File Format

ID	EVID	AMT	TIME	DV	MDV	CENS	GROUP	SEX	OCC
1	4	10000	0	.	1	0	VD3 Suf	M	1
1	0	.	0.5	270.65	0	0	VD3 Suf	M	1
1	0	.	4	89.88	0	0	VD3 Suf	M	1
1	0	.	8	30.721	0	0	VD3 Suf	M	1
1	4	10000	0	.	1	0	VD3 Suf	M	2
1	0	.	0.5	212.25	0	0	VD3 Suf	M	2
1	0	.	4	58.696	0	0	VD3 Suf	M	2
1	0	.	8	16.431	0	0	VD3 Suf	M	2
2	4	10000	0	.	1	0	VD3 Suf	M	1
2	0	.	0.5	82.77	0	0	VD3 Suf	M	1
2	0	.	4	146.77	0	0	VD3 Suf	M	1
2	0	.	8	73.025	0	0	VD3 Suf	M	1
2	4	10000	0	.	1	0	VD3 Suf	M	2
2	0	.	0.5	315.57	0	0	VD3 Suf	M	2
2	0	.	4	194.31	0	0	VD3 Suf	M	2
2	0	.	8	20.446	0	0	VD3 Suf	M	2
3	4	10000	0	.	1	0	VD3 Suf	M	1
3	0	.	0.5	208.46	0	0	VD3 Suf	M	1
3	0	.	4	103.68	0	0	VD3 Suf	M	1
3	0	.	8	26.015	0	0	VD3 Suf	M	1
3	4	10000	0	.	1	0	VD3 Suf	M	2
3	0	.	0.5	207.19	0	0	VD3 Suf	M	2
3	0	.	4	89.2	0	0	VD3 Suf	M	2
3	0	.	8	10.361	0	0	VD3 Suf	M	2
4	4	10000	0	.	1	0	VD3 Suf	F	1
4	0	.	0.5	231.05	0	0	VD3 Suf	F	1
4	0	.	4	54.116	0	0	VD3 Suf	F	1
4	0	.	8	22.004	0	0	VD3 Suf	F	1
4	4	10000	0	.	1	0	VD3 Suf	F	2
4	0	.	0.5	136.64	0	0	VD3 Suf	F	2
4	0	.	4	63.994	0	0	VD3 Suf	F	2
4	0	.	8	10.774	0	0	VD3 Suf	F	2
5	4	10000	0	.	1	0	VD3 Suf	F	1
5	0	.	0.5	144.41	0	0	VD3 Suf	F	1
5	0	.	4	105.56	0	0	VD3 Suf	F	1
5	0	.	8	11.198	0	0	VD3 Suf	F	1

5	4	10000	0	.	1	0	VD3 Suf	F	2
5	0	.	0.5	124.69	0	0	VD3 Suf	F	2
5	0	.	4	72.682	0	0	VD3 Suf	F	2
5	0	.	8	10.156	0	0	VD3 Suf	F	2
6	4	10000	0	.	1	0	VD3 Suf	F	1
6	0	.	0.5	217.29	0	0	VD3 Suf	F	1
6	0	.	4	62.725	0	0	VD3 Suf	F	1
6	0	.	8	14.683	0	0	VD3 Suf	F	1
6	4	10000	0	.	1	0	VD3 Suf	F	2
6	0	.	0.5	151.83	0	0	VD3 Suf	F	2
6	0	.	4	42.565	0	0	VD3 Suf	F	2
6	0	.	8	25.354	0	0	VD3 Suf	F	2
7	4	10000	0	.	1	0	VD3 Def	M	1
7	0	.	0.5	194.4	0	0	VD3 Def	M	1
7	0	.	4	87.779	0	0	VD3 Def	M	1
7	0	.	8	16.22	0	0	VD3 Def	M	1
7	4	10000	0	.	1	0	VD3 Def	M	2
7	0	.	0.5	177.81	0	0	VD3 Def	M	2
7	0	.	4	83.212	0	0	VD3 Def	M	2
7	0	.	8	15.523	0	0	VD3 Def	M	2
8	4	10000	0	.	1	0	VD3 Def	M	1
8	0	.	0.5	240.61	0	0	VD3 Def	M	1
8	0	.	4	99.618	0	0	VD3 Def	M	1
8	0	.	8	11.773	0	0	VD3 Def	M	1
8	4	10000	0	.	1	0	VD3 Def	M	2
8	0	.	0.5	117.58	0	0	VD3 Def	M	2
8	0	.	4	62.561	0	0	VD3 Def	M	2
8	0	.	8	13.735	0	0	VD3 Def	M	2
9	4	10000	0	.	1	0	VD3 Def	M	1
9	0	.	0.5	267.78	0	0	VD3 Def	M	1
9	0	.	4	81.306	0	0	VD3 Def	M	1
9	0	.	8	10.821	0	0	VD3 Def	M	1
9	4	10000	0	.	1	0	VD3 Def	M	2
9	0	.	0.5	209.13	0	0	VD3 Def	M	2
9	0	.	4	70.903	0	0	VD3 Def	M	2
9	0	.	8	13.061	0	0	VD3 Def	M	2
10	4	10000	0	.	1	0	VD3 Def	F	1
10	0	.	0.5	149.17	0	0	VD3 Def	F	1
10	0	.	4	100.34	0	0	VD3 Def	F	1

10	0	.	8	25.171	0	0	VD3 Def	F	1
10	4	10000	0	.	1	0	VD3 Def	F	2
10	0	.	0.5	187.75	0	0	VD3 Def	F	2
10	0	.	4	195.94	0	0	VD3 Def	F	2
10	0	.	8	55.501	0	0	VD3 Def	F	2
11	4	10000	0	.	1	0	VD3 Def	F	1
11	0	.	0.5	206.1	0	0	VD3 Def	F	1
11	0	.	4	168.82	0	0	VD3 Def	F	1
11	0	.	8	33.172	0	0	VD3 Def	F	1
11	4	10000	0	.	1	0	VD3 Def	F	2
11	0	.	0.5	128.51	0	0	VD3 Def	F	2
11	0	.	4	81.369	0	0	VD3 Def	F	2
11	0	.	8	13.53	0	0	VD3 Def	F	2
12	4	10000	0	.	1	0	VD3 Def	F	1
12	0	.	0.5	158.35	0	0	VD3 Def	F	1
12	0	.	4	63.047	0	0	VD3 Def	F	1
12	0	.	8	43.592	0	0	VD3 Def	F	1
12	4	10000	0	.	1	0	VD3 Def	F	2
12	0	.	0.5	161.37	0	0	VD3 Def	F	2
12	0	.	4	41.635	0	0	VD3 Def	F	2
12	0	.	8	52.45	0	0	VD3 Def	F	2
13	4	10000	0	.	1	0	VD3 Suf	M	1
13	0	.	0.25	109.71	0	0	VD3 Suf	M	1
13	0	.	2	350.04	0	0	VD3 Suf	M	1
13	0	.	24	3.9807	0	0	VD3 Suf	M	1
13	4	10000	0	.	1	0	VD3 Suf	M	2
13	0	.	0.25	107.39	0	0	VD3 Suf	M	2
13	0	.	2	366.26	0	0	VD3 Suf	M	2
13	0	.	24	1.8802	0	0	VD3 Suf	M	2
14	4	10000	0	.	1	0	VD3 Suf	M	1
14	0	.	0.25	87.715	0	0	VD3 Suf	M	1
14	0	.	2	500	0	-1	VD3 Suf	M	1
14	0	.	24	1.9021	0	0	VD3 Suf	M	1
14	4	10000	0	.	1	0	VD3 Suf	M	2
14	0	.	0.25	101.99	0	0	VD3 Suf	M	2
14	0	.	2	297.27	0	0	VD3 Suf	M	2
14	0	.	24	3.3895	0	0	VD3 Suf	M	2
15	4	10000	0	.	1	0	VD3 Suf	M	1
15	0	.	0.25	117.65	0	0	VD3 Suf	M	1

15	0	.	2	483.94	0	0	VD3 Suf	M	1
15	0	.	24	9.8162	0	0	VD3 Suf	M	1
15	4	10000	0	.	1	0	VD3 Suf	M	2
15	0	.	0.25	116.5	0	0	VD3 Suf	M	2
15	0	.	2	320.75	0	0	VD3 Suf	M	2
15	0	.	24	3.3706	0	0	VD3 Suf	M	2
16	4	10000	0	.	1	0	VD3 Suf	F	1
16	0	.	0.25	244.67	0	0	VD3 Suf	F	1
16	0	.	2	500	0	-1	VD3 Suf	F	1
16	0	.	24	10.835	0	0	VD3 Suf	F	1
16	4	10000	0	.	1	0	VD3 Suf	F	2
16	0	.	0.25	410.04	0	0	VD3 Suf	F	2
16	0	.	2	395.48	0	0	VD3 Suf	F	2
16	0	.	24	14.402	0	0	VD3 Suf	F	2
17	4	10000	0	.	1	0	VD3 Suf	F	1
17	0	.	0.25	197.71	0	0	VD3 Suf	F	1
17	0	.	2	405.32	0	0	VD3 Suf	F	1
17	0	.	24	2.5167	0	0	VD3 Suf	F	1
17	4	10000	0	.	1	0	VD3 Suf	F	2
17	0	.	0.25	126.39	0	0	VD3 Suf	F	2
17	0	.	2	441.19	0	0	VD3 Suf	F	2
17	0	.	24	1.8978	0	0	VD3 Suf	F	2
18	4	10000	0	.	1	0	VD3 Suf	F	1
18	0	.	0.25	167.43	0	0	VD3 Suf	F	1
18	0	.	2	466.97	0	0	VD3 Suf	F	1
18	0	.	24	1.6688	0	0	VD3 Suf	F	1
18	4	10000	0	.	1	0	VD3 Suf	F	2
18	0	.	0.25	183.93	0	0	VD3 Suf	F	2
18	0	.	2	295.49	0	0	VD3 Suf	F	2
18	0	.	24	1.8468	0	0	VD3 Suf	F	2
19	4	10000	0	.	1	0	VD3 Def	M	1
19	0	.	0.25	144.72	0	0	VD3 Def	M	1
19	0	.	2	500	0	-1	VD3 Def	M	1
19	0	.	24	6.387	0	0	VD3 Def	M	1
19	4	10000	0	.	1	0	VD3 Def	M	2
19	0	.	0.25	165.73	0	0	VD3 Def	M	2
19	0	.	2	286.95	0	0	VD3 Def	M	2
19	0	.	24	2.3326	0	0	VD3 Def	M	2
20	4	10000	0	.	1	0	VD3 Def	M	1

20	0	.	0.25	143.39	0	0	VD3 Def	M	1
20	0	.	2	760.24	0	0	VD3 Def	M	1
20	0	.	24	7.0494	0	0	VD3 Def	M	1
20	4	10000	0	.	1	0	VD3 Def	M	2
20	0	.	0.25	263.27	0	0	VD3 Def	M	2
20	0	.	2	376.78	0	0	VD3 Def	M	2
20	0	.	24	1	0	1	VD3 Def	M	2
21	4	10000	0	.	1	0	VD3 Def	M	1
21	0	.	0.25	275.48	0	0	VD3 Def	M	1
21	0	.	2	500	0	-1	VD3 Def	M	1
21	0	.	24	5.7321	0	0	VD3 Def	M	1
21	4	10000	0	.	1	0	VD3 Def	M	2
21	0	.	0.25	481.28	0	0	VD3 Def	M	2
21	0	.	2	500	0	-1	VD3 Def	M	2
21	0	.	24	8.2798	0	0	VD3 Def	M	2
22	4	10000	0	.	1	0	VD3 Def	F	1
22	0	.	0.25	241.55	0	0	VD3 Def	F	1
22	0	.	2	434.94	0	0	VD3 Def	F	1
22	0	.	24	4.3092	0	0	VD3 Def	F	1
22	4	10000	0	.	1	0	VD3 Def	F	2
22	0	.	0.25	145.66	0	0	VD3 Def	F	2
22	0	.	2	212.54	0	0	VD3 Def	F	2
22	0	.	24	4.7125	0	0	VD3 Def	F	2
23	4	10000	0	.	1	0	VD3 Def	F	1
23	0	.	0.25	36.55	0	0	VD3 Def	F	1
23	0	.	2	500	0	-1	VD3 Def	F	1
23	0	.	24	10.186	0	0	VD3 Def	F	1
23	4	10000	0	.	1	0	VD3 Def	F	2
23	0	.	0.25	240.93	0	0	VD3 Def	F	2
23	0	.	2	436.13	0	0	VD3 Def	F	2
23	0	.	24	15.418	0	0	VD3 Def	F	2
24	4	10000	0	.	1	0	VD3 Def	F	1
24	0	.	0.25	94.266	0	0	VD3 Def	F	1
24	0	.	2	323.65	0	0	VD3 Def	F	1
24	0	.	24	1.6483	0	0	VD3 Def	F	1
24	4	10000	0	.	1	0	VD3 Def	F	2
24	0	.	0.25	155.4	0	0	VD3 Def	F	2
24	0	.	2	250.23	0	0	VD3 Def	F	2
24	0	.	24	1.9454	0	0	VD3 Def	F	2

25	4	10000	0	.	1	0	VD3 Suf	M	1
25	0	.	0.125	49.421	0	0	VD3 Suf	M	1
25	0	.	1	951	0	0	VD3 Suf	M	1
25	0	.	16	5.4264	0	0	VD3 Suf	M	1
25	4	10000	0	.	1	0	VD3 Suf	M	2
25	0	.	0.125	24.172	0	0	VD3 Suf	M	2
25	0	.	1	500	0	-1	VD3 Suf	M	2
25	0	.	16	2.5952	0	0	VD3 Suf	M	2
26	4	10000	0	.	1	0	VD3 Suf	M	1
26	0	.	0.125	35.897	0	0	VD3 Suf	M	1
26	0	.	1	451.17	0	0	VD3 Suf	M	1
26	0	.	16	3.4341	0	0	VD3 Suf	M	1
26	4	10000	0	.	1	0	VD3 Suf	M	2
26	0	.	0.125	82.069	0	0	VD3 Suf	M	2
26	0	.	1	500	0	-1	VD3 Suf	M	2
26	0	.	16	16.811	0	0	VD3 Suf	M	2
27	4	10000	0	.	1	0	VD3 Suf	M	1
27	0	.	0.125	45.322	0	0	VD3 Suf	M	1
27	0	.	1	806.88	0	0	VD3 Suf	M	1
27	0	.	16	17.046	0	0	VD3 Suf	M	1
27	4	10000	0	.	1	0	VD3 Suf	M	2
27	0	.	0.125	177.03	0	0	VD3 Suf	M	2
27	0	.	1	500	0	-1	VD3 Suf	M	2
27	0	.	16	18.329	0	0	VD3 Suf	M	2
28	4	10000	0	.	1	0	VD3 Suf	F	1
28	0	.	0.125	116.65	0	0	VD3 Suf	F	1
28	0	.	1	448.38	0	0	VD3 Suf	F	1
28	0	.	16	2.0614	0	0	VD3 Suf	F	1
28	4	10000	0	.	1	0	VD3 Suf	F	2
28	0	.	0.125	23.52	0	0	VD3 Suf	F	2
28	0	.	1	500	0	-1	VD3 Suf	F	2
28	0	.	16	8.1466	0	0	VD3 Suf	F	2
29	4	10000	0	.	1	0	VD3 Suf	F	1
29	0	.	0.125	77.332	0	0	VD3 Suf	F	1
29	0	.	1	733.56	0	0	VD3 Suf	F	1
29	0	.	16	1.9898	0	0	VD3 Suf	F	1
29	4	10000	0	.	1	0	VD3 Suf	F	2
29	0	.	0.125	56.101	0	0	VD3 Suf	F	2
29	0	.	1	500	0	-1	VD3 Suf	F	2

29	0	.	16	9.1881	0	0	VD3 Suf	F	2
30	4	10000	0	.	1	0	VD3 Suf	F	1
30	0	.	0.125	54.215	0	0	VD3 Suf	F	1
30	0	.	1	211.95	0	0	VD3 Suf	F	1
30	0	.	16	3.161	0	0	VD3 Suf	F	1
30	4	10000	0	.	1	0	VD3 Suf	F	2
30	0	.	0.125	69.153	0	0	VD3 Suf	F	2
30	0	.	1	500	0	-1	VD3 Suf	F	2
30	0	.	16	6.2417	0	0	VD3 Suf	F	2
31	4	10000	0	.	1	0	VD3 Def	M	1
31	0	.	0.125	25.956	0	0	VD3 Def	M	1
31	0	.	1	1083.6	0	0	VD3 Def	M	1
31	0	.	16	6.621	0	0	VD3 Def	M	1
31	4	10000	0	.	1	0	VD3 Def	M	2
31	0	.	0.125	24.375	0	0	VD3 Def	M	2
31	0	.	1	500	0	-1	VD3 Def	M	2
31	0	.	16	11.12	0	0	VD3 Def	M	2
32	4	10000	0	.	1	0	VD3 Def	M	1
32	0	.	0.125	36.307	0	0	VD3 Def	M	1
32	0	.	1	205.57	0	0	VD3 Def	M	1
32	0	.	16	7.1303	0	0	VD3 Def	M	1
32	4	10000	0	.	1	0	VD3 Def	M	2
32	0	.	0.125	22.768	0	0	VD3 Def	M	2
32	0	.	1	500	0	-1	VD3 Def	M	2
32	0	.	16	8.5466	0	0	VD3 Def	M	2
33	4	10000	0	.	1	0	VD3 Def	M	1
33	0	.	0.125	77.736	0	0	VD3 Def	M	1
33	0	.	1	383.02	0	0	VD3 Def	M	1
33	0	.	16	2.6046	0	0	VD3 Def	M	1
33	4	10000	0	.	1	0	VD3 Def	M	2
33	0	.	0.125	45.897	0	0	VD3 Def	M	2
33	0	.	1	500	0	-1	VD3 Def	M	2
33	0	.	16	7.4123	0	0	VD3 Def	M	2
34	4	10000	0	.	1	0	VD3 Def	F	1
34	0	.	0.125	30.864	0	0	VD3 Def	F	1
34	0	.	1	277.73	0	0	VD3 Def	F	1
34	0	.	16	4.0154	0	0	VD3 Def	F	1
34	4	10000	0	.	1	0	VD3 Def	F	2
34	0	.	0.125	48.964	0	0	VD3 Def	F	2

34	0	.	1	173.95	0	0	VD3 Def	F	2
34	0	.	16	4.3532	0	0	VD3 Def	F	2
35	4	10000	0	.	1	0	VD3 Def	F	1
35	0	.	0.125	77.805	0	0	VD3 Def	F	1
35	0	.	1	457.04	0	0	VD3 Def	F	1
35	0	.	16	2.7889	0	0	VD3 Def	F	1
35	4	10000	0	.	1	0	VD3 Def	F	2
35	0	.	0.125	29.279	0	0	VD3 Def	F	2
35	0	.	1	149.3	0	0	VD3 Def	F	2
35	0	.	16	6.2042	0	0	VD3 Def	F	2
36	4	10000	0	.	1	0	VD3 Def	F	1
36	0	.	0.125	500	0	-1	VD3 Def	F	1
36	0	.	1	404.1	0	0	VD3 Def	F	1
36	0	.	16	2.9067	0	0	VD3 Def	F	1
36	4	10000	0	.	1	0	VD3 Def	F	2
36	0	.	0.125	44.718	0	0	VD3 Def	F	2
36	0	.	1	500	0	-1	VD3 Def	F	2
36	0	.	16	7.8308	0	0	VD3 Def	F	2
