# Supplementary figures and images for: Vitamin D levels do not cause vitamin-drug interactions with dexamethasone or dasatinib in mice
Source: PLoS One. 2021 Oct 20;16(10):e0258579. doi: 10.1371/journal.pone.0258579 (PMC8528301; doi:10.1371/journal.pone.0258579)

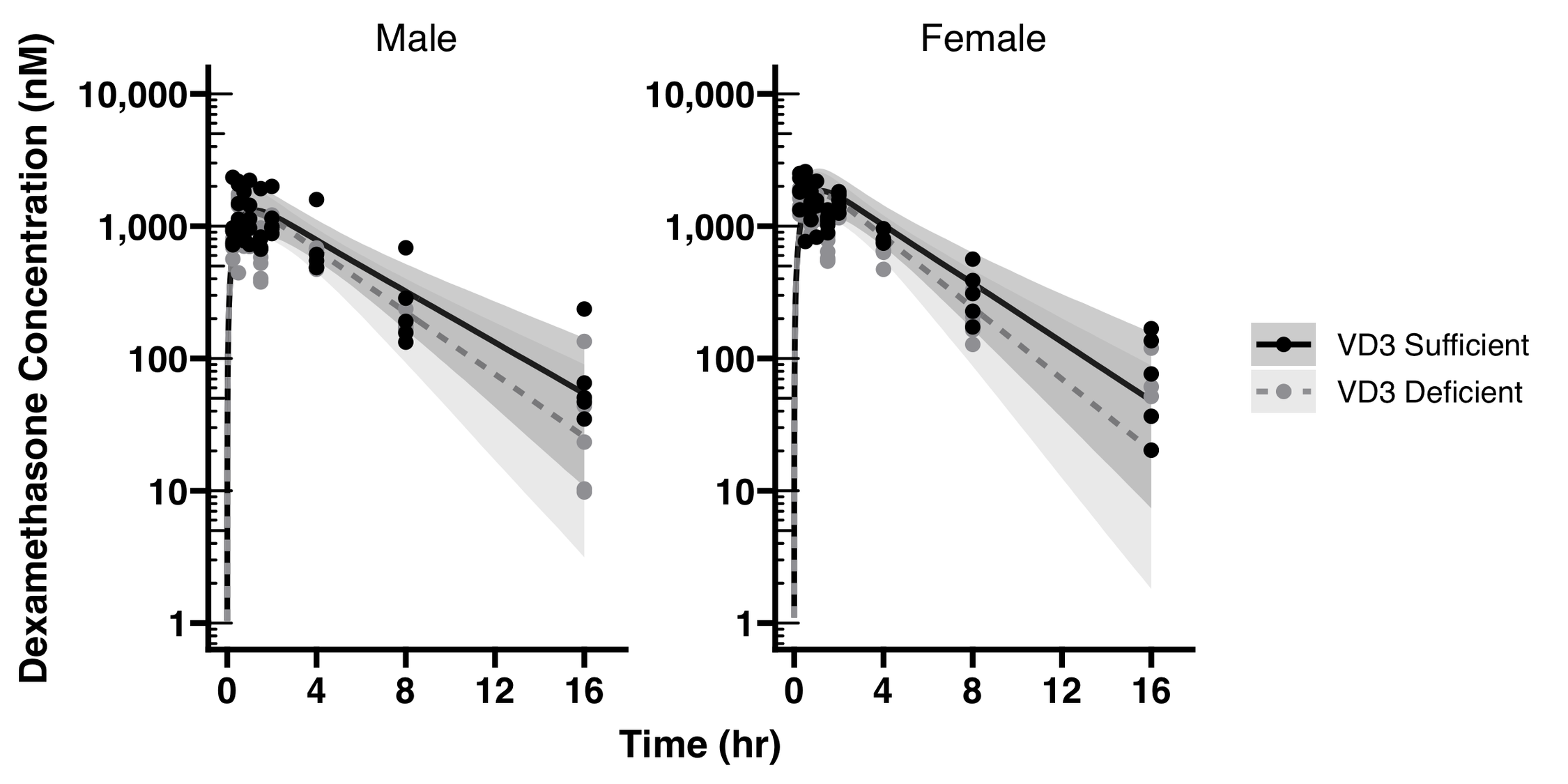

Supplement: S1 Fig — Observed and model predicted dexamethasone plasma concentration vs. time (Ct) profiles by sex (panels) and VD3 status. The black solid (VD3 Sufficient) and gray dashed (VD3 Deficient) lines indicate the population model predicted median concentrations, whereas the shaded areas represent the model’s 90% prediction intervals (90% PI) by VD3 status. There were statistically significant differences in dexamethasone PK between sexes, with males having approximately 25% lower AUC values (p<0.05). VD3 deficiency also resulted in an approximately 25% lower AUC value across both sexes (p<0.05). (TIF) [file pone.0258579.s002.tif]
